# Pink-Colored Grape Berry Is the Result of Short Insertion in Intron of Color Regulatory Gene

**DOI:** 10.1371/journal.pone.0021308

**Published:** 2011-06-17

**Authors:** Mamiko Shimazaki, Keiko Fujita, Hironori Kobayashi, Shunji Suzuki

**Affiliations:** 1 The Institute of Enology and Viticulture, University of Yamanashi, Kofu, Yamanashi, Japan; 2 Product Development Research Laboratory, Mercian Corporation, Fujisawa, Kanagawa, Japan; Tulane University Health Sciences Center, United States of America

## Abstract

We report here that pink grape berries were obtained by a short insertion in the intron of the *MybA1* gene, a gene that regulates grape berry color. Genetic variation was detected among the *MybA1* genes from grapes cultivated worldwide. PCR analysis of the *MybA1* gene demonstrated that the size of the *MybA1* gene in the red allele differs among grapes. Oriental *V. vinifera* bearing pink berries has the longest *MybA1* gene among grapes, whereas the shortest *MybA1* gene was detected in occidental *V. vinifera* grapes. The nucleotide sequences of the *MybA1* genes demonstrated that oriental *V. vinifera* has two additional gene fragments (44 bp and 111 bp) in the promoter region of the *MybA1* gene in the red allele and another 33 bp fragment in the second intron of the *MybA1* gene in the red allele. The short insertion in the intron decreased the transcription activity in the model system and retained *MybA1* transcripts with unspliced intron in the total RNA. From the experiments using deletion mutants of the 33 bp short insertion, 16 bp of the 3′ end in the insertion is a key structure for a defect in splicing of *MybA1* transcripts. Thus, a weakly colored grape berry might be a result of the short insertion in the intron of a color regulatory gene. This is new evidence concerning the molecular mechanism of the fate of grape berry color. These findings are expected to contribute to the further understanding of the color variation in grape berries, which is correlated with the evolutional events occurring in the *MybA1* gene of grapes.

## Introduction

Wine grapes belong to the species *Vitis vinifera*, which is classified into three groups: *occidentalis*, *pontica*, and *orientalis* [1]. The East Asian *V. vinifera*, including the Japanese and Chinese cultivars, is grouped in *orientalis* as well as the West Asian *V. vinifera* cultivars, including Muscat of Alexandria and Sultanina [2]. The Japanese and Chinese *V. vinifera* cultivars have unique characteristics that distinguish them from occidental *V. vinifera*. For example, the cluster and berry sizes of the indigenous Japanese *V. vinifera* cultivar Koshu and the indigenous Chinese *V. vinifera* cultivar Ryugan are two to three times larger than those of the major occidental cultivars, such as Cabernet Sauvignon, Pinot Noir, and Chardonnay. One of unique phenomena in those berries is that their skin colors are confined to green-yellow or pink. Fundamental questions associated with skin color deficiency in Asian *V. vinifera* remain unanswered, although the pink-colored berry skin of Koshu is due to the low accumulation of anthocyanins in the skin [3].

Black/red berries are the result of anthocyanin accumulation in the skins. UDP-glucose:flavonoid 3-o-glucosyltransferase (UFGT), which glycosylates anthocyanidins, is the key enzyme responsible for the accumulation of anthocyanins in grape berry skins [4] (Figure S1). Its expression is transcriptionally regulated by MybA transcription factors [5], [6]. The *MybA* genes are closely clustered in a single locus, referred to as the berry color locus [6]. The insertion of the retrotransposon *Gret1* into the promoter region of the *MybA1* gene induced the inactivation of the promoter, resulting in the white berry allele [5]. On the other hand, nucleotide mutations in the *MybA2* gene produced the white berry allele of the *MybA2* gene [6]. A large deletion of the *MybA* genes in the red allele at the berry color locus also induced the white mutation of berry skins [7]–[9]. Thus, the mutation in the *MybA* genes is a good basis upon which to understand the genetic modifications that cause phenotypic mutations in berry color. Although the precise molecular mechanism of the somatic mutation at the red color locus remains unclear, the *MybA* cluster may be preferentially fragile in the grape genomic sequence and the somatic mutations have frequently occurred, resulting in frequent color mutation.

To determine why Japanese and Chinese grape berries accumulate less anthocyanin in their skins than black/red occidental grape berries, we performed a comparative genomic analysis among *Vitis* species. In the present study, we identified three distinctive fragments in the *MybA1* genes of oriental *V. vinifera*. One fragment inserted in the second intron of Koshu *MybA1* gene decreased *MybA1* transcription. Consequently, we propose a new hypothesis concerning the molecular mechanism of the fate of grape berry color.

## Results

### Anthocyanin accumulation in Koshu grape berry

**Figure 1:**
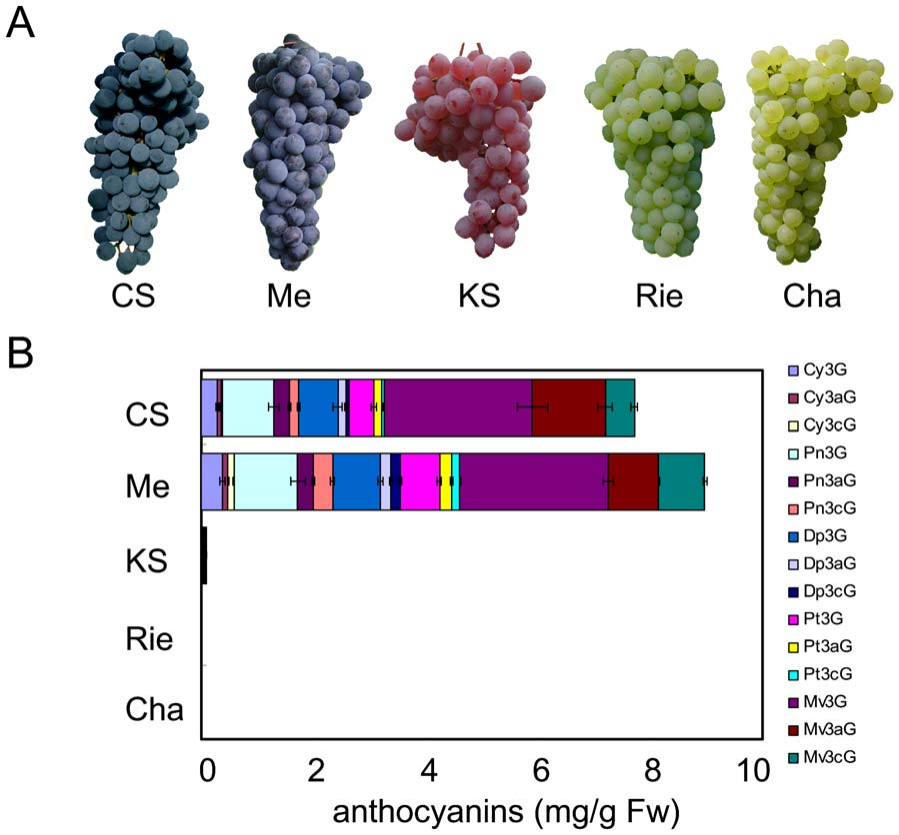
Anthocyanins in berry skin of grapes. (A) Features of bunches. (B) Total amounts of anthocyanins. Measurement of anthocyanins was performed as described in Materials and Methods.Experimental Procedures. Bars indicate means ± standard deviations of duplicate experiments. CS: Cabernet Sauvignon, Me: Merlot, KS: Koshu, Rie: Riesling, Cha: Chardonnay.

The biosynthetic pathway of anthocyanins in grapes is shown in Figure S1. The concentrations of anthocyanins in the skins of black grape berries, Cabernet Sauvignon and Merlot, were higher than those in the skins of pink grape berry, Koshu, and white grape berries, Riesling and Chardonnay, correlating with berry color (Figure 1).

### Low *MybA1* gene expression is associated with pink color of Koshu grape berry

**Figure 2:**
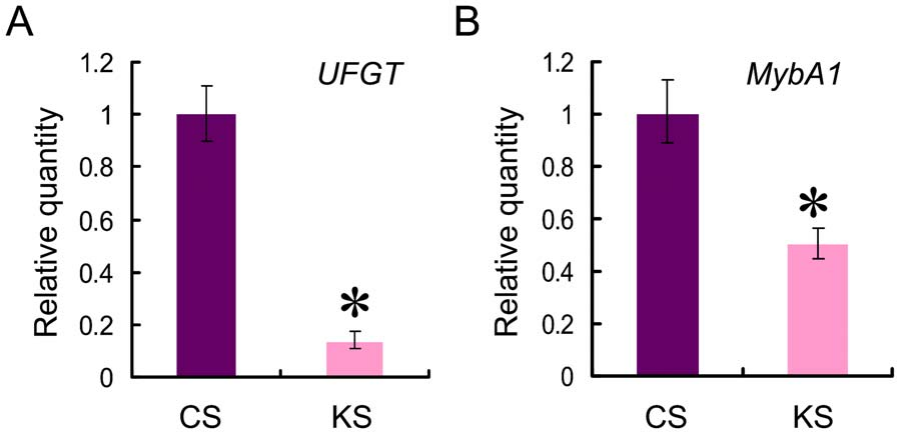
*UFGT* and *MybA1* gene expression in Koshu grape berry skin. (A) *UFGT*. (B) *MybA1*. CS, Cabernet Sauvignon. KS, Koshu. *p<0.01 as compared with CS.

MybA1 regulates anthocyanin accumulation in grape berry skin via *UFGT* gene expression (Figure S1). Koshu berry skin expressed low levels of both *UFGT* and *MybA1* genes at harvest compared with Cabernet Sauvignon berry skin (Figure 2). These findings suggest that the low expression of the *MybA1* gene in Koshu berry skin is associated with the pink color of the skin.

### Analysis of *MybA1* gene structure in Koshu

**Figure 3:**
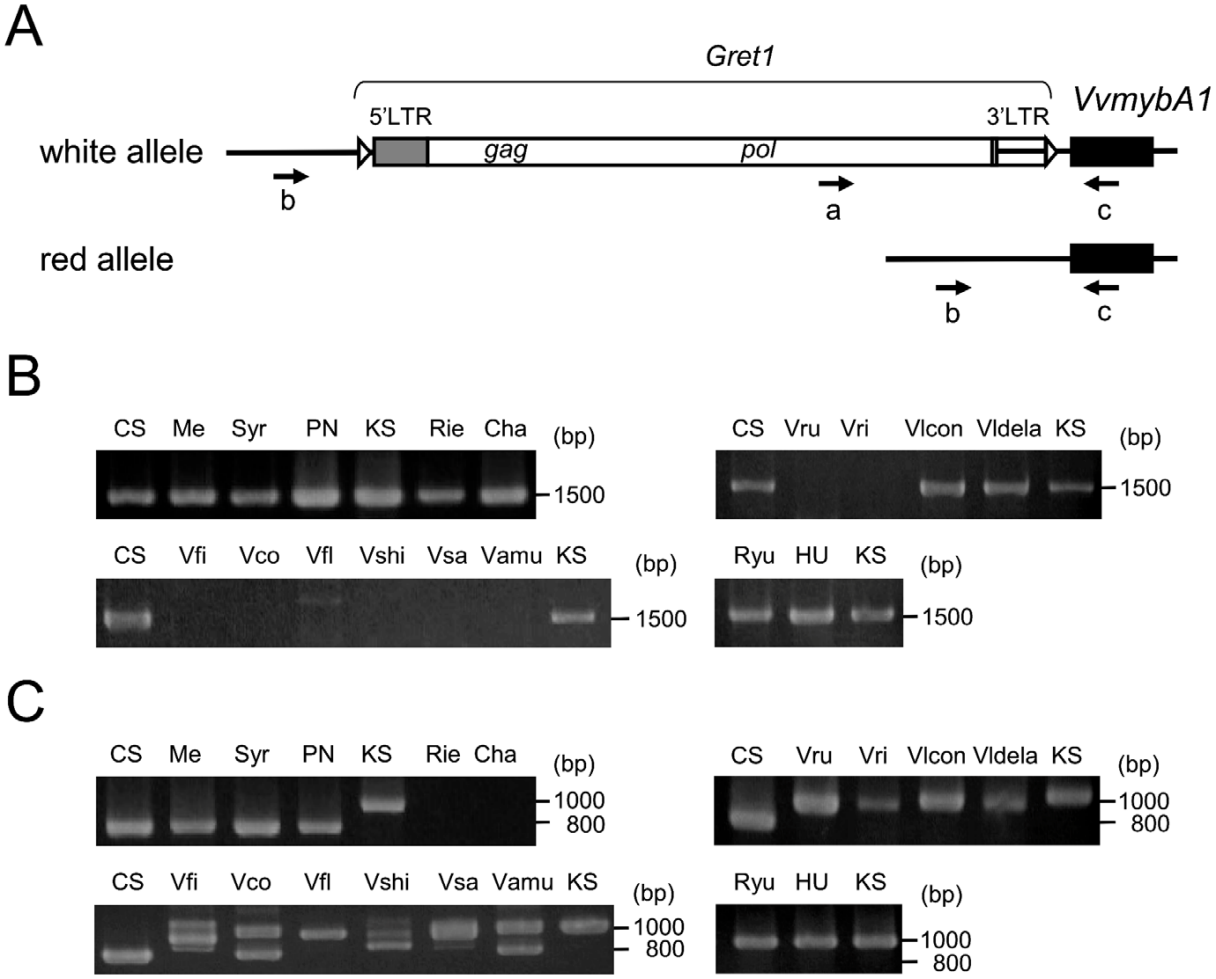
Analysis of *MybA1* gene structure in grapes. (A) Primer maps used for PCR analysis. (B) PCR analysis of white alleles in *Vitis* species using primer set a and c. (C) PCR analysis of red alleles in *Vitis* species using primer set b and c. Numbers on the right indicate the positions of the molecular size markers. These results represent reproducible data from three independent experiments. CS, Cabernet Sauvignon. Me, Merlot. Syr, Syrah. PN, Pinot Noir. KS, Koshu. Rie, Riesling. Cha, Chardonnay. Ryu, Ryugan. HU, Huotianhong. Vru, *V. rupestris*. Vri, *V. riparia*. Vlcon, *V. labrusca* cv. Concord. Vldela, *V. labruscana* cv. Delaware. Vfi, *V. ficifolia*. Vco, *V. coignetiae*. Vfl, *V. flexuosa*. Vshi, *V. shiragai*. Vsa, *V. saccharifera*. Vamu, *V. amurensis*.

**Figure 4:**
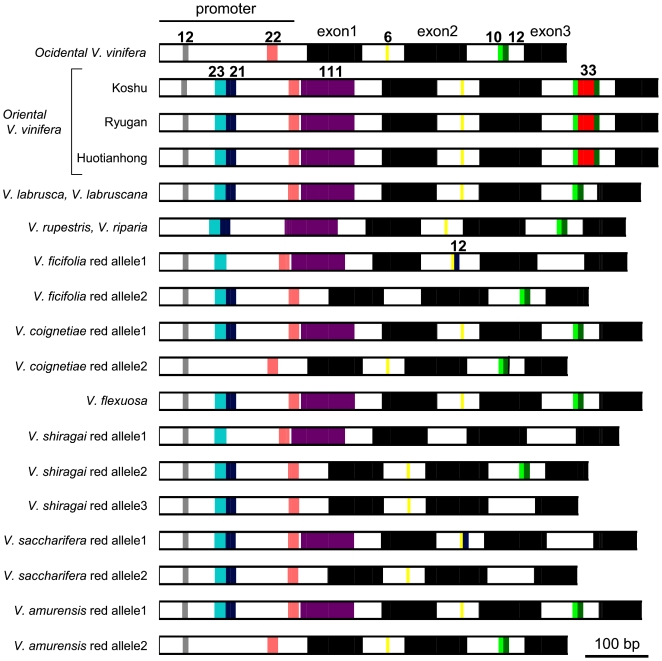
Comparison of *MybA1* gene structure in *Vitis* grapes. Black boxes indicate three exons in the *MybA1* gene. Other colored boxes indicate unique gene fragments in each *MybA1* gene sequence. The same colored box indicates the same gene fragment. Numbers on the boxes indicate the base pairs in each gene fragment. Single-nucleotide polymorphisms among *Vitis* species are not shown.

PCR primer set a and c was used to amplify the region between the *MybA1* coding region and *Gret1* in the white allele, and primer set b and c was used to amplify the region between the *MybA1* coding region and its promoter in the red allele [5] (Figure 3A). White alleles were detected in black, pink, and white *V. vinifera* (Figure 3B), while the red allele was not detected in white *V. vinifera* cvs. Riesling and Chardonnay (Figure 3C, Rie and Cha). These results coincide with those of a previous study [5] and suggest that black/red cultivars have red and white alleles in their genome whereas white cultivars have only white alleles in their genome. Pink Koshu had the largest *MybA1* gene among the *V. vinifera* species (Figure 3C, KS). Interestingly, the red allele of oriental *V. vinifera* cvs. Ryugan and Huotianhong also had a large *MybA1* gene (Figure 3C, Rhu and HU). The PCR product amplified from the red allele of oriental *V. vinifera* was approximately 200 bp larger than those of the occidental *V. vinifera* cultivars (Figure 3C, Table S1). DNA sequence analysis of the *MybA1* genes demonstrated that Koshu had three additional gene fragments (44 bp, 111 bp, and 33 bp) in the red allele of the *MybA*1 gene relative to the sequence of Cabernet Sauvignon (Figure S2A). Two of the fragments (44 bp and 111 bp) occur were found in the promoter region of the *MybA1* gene and the other 33 bp fragment was found in the second intron of the *MybA1* gene (Figure 4, Figure S2A). Without exception, oriental *V. vinifera* bearing pink berries had the fragments in the *MybA1* gene of the red allele (Figure 4, Figure S2A). Meanwhile, the DNA sequence of the *MybA1* gene of the white allele was identical between Koshu and Cabernet Sauvignon (Figure S2B). These results suggest that the *MybAl* gene in the red allele of *V. vinifera orientalis* is structurally different from that of *V. vinifera occidentalis*.

### Analysis of the *MybA1* gene structure in grapes

The *Gret1*-inserted *MybA1* white allele was also detected in *V. labrusca* and *V. labruscana* (Figure 3B, Vlcon and Vldela). In contrast, the North American rootstock *Vitis* species, *V. rupestris* and *V. riparia*, and the East Asian *Vitis* species, *V. ficifolia*, *V. coignetiae*, *V. flexuosa*, *V. shiragai*, *V. saccharifera*, and *V. amurensis*, did not contain the *Gret1* insertion in the *MybA1* gene (Figure 3B). These results are supported by a previous report [10]. North American *Vitis* species generated a single amplified product on PCR with the primer set b and c, although the sizes of the products differed among the *Vitis* species (Figure 3C, Vru, Vri, Vlcon, and Vldela). This primer set amplified two or three products from the East Asian *Vitis* species, except *V. flexuosa* (Figure 3C). Together with the fact that the East Asian *Vitis* species have no white-allele-containing *Gret1* retrotransposon, these results suggest that the East Asian *Vitis* species contain only the red *MybA1* alleles.

Two fragments inserted into the promoter region of the *MybA1* gene of *V. vinifera orientalis* were also detected in the *MybA1* genes of the North American and East Asian *Vitis* species (Figures 4, Figure S3). In contrast, the 33 bp fragment in the second intron was distinctive of *V. vinifera orientalis,* such as Koshu, Ryugan, and Huotianhong, all of which bear pink berries (Figures 4, Figure S3).

Cluster analysis of the *MybA1* genes among grapes demonstrated that *V. vinifera orientalis* cultivars were far away from *V. vinifera occidentalis* cultivars (Figure S4). Again, the *MybAl* gene in the red allele of *V. vinifera orientalis* is genetically different from that of *V. vinifera occidentalis*.

### The 33 bp short insertion decreases the amount of transcripts

**Figure 5:**
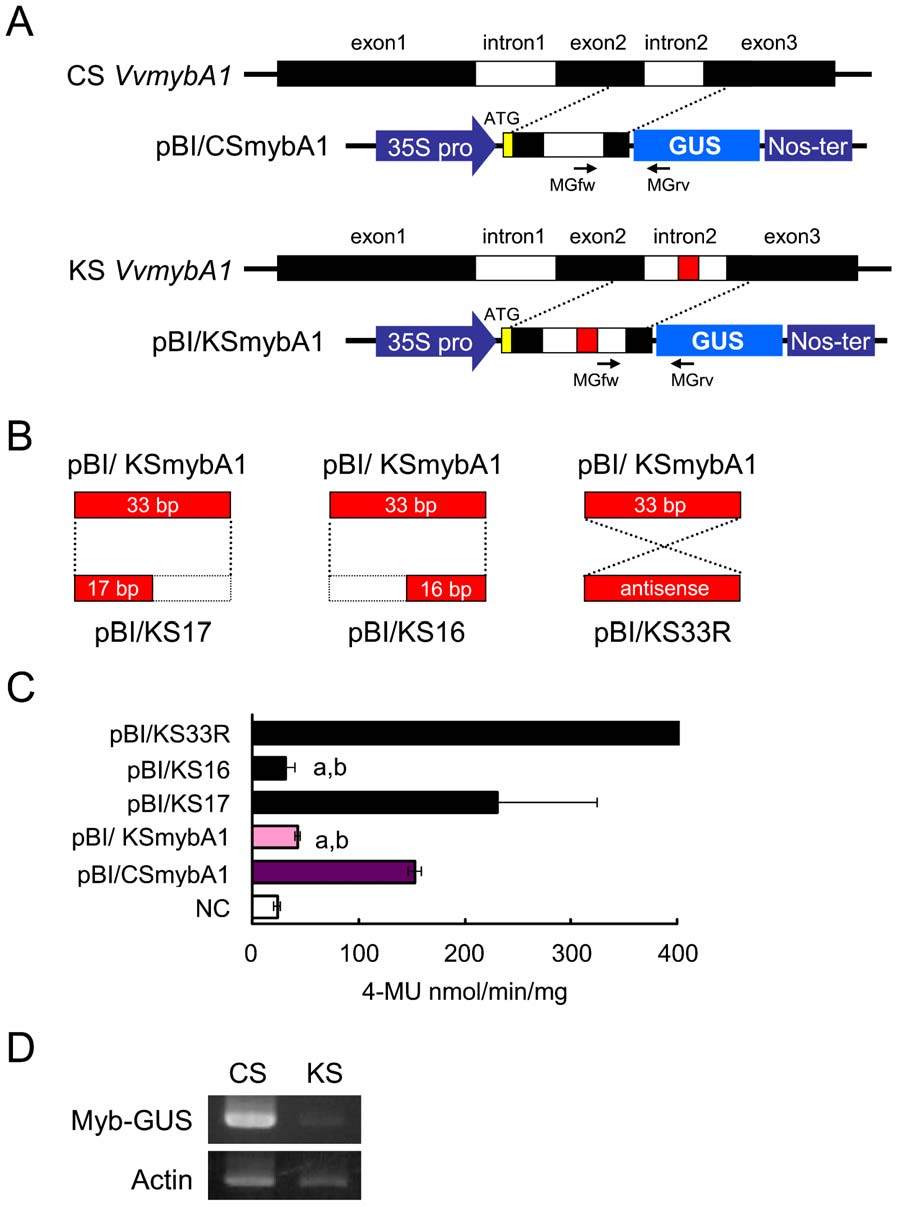
Effect of 33 bp short insertion on the transcription of a reporter gene. (A) Transformation constructs. Gene fragments from the second exon to the third exon, including the second intron, of Cabernet Sauvignon or Koshu *MybA1* gene were inserted in front of the GUS reporter gene, resulting in pBI/CSmybA1 or pBI/KSmybA1 plant expression plasmids, respectively. Red boxes indicate the 33 bp short insertion in the second intron of Koshu *MybA1*. Yellow boxes indicate the ATG start codon. 35S pro, 35S promoter. Nos-ter, Nos terminator. (B) A schematic representation of the deletion (pBI/KS17 and pBI/KS16) and antisense (pBI/KS33R) mutants for 33 bp short insertion. The plant expression vectors having these mutants were constructed by procedures detailed in Methods. (C) GUS activity. BY-2 cells transformed by the plant expression vectors were used. GUS activity in the graph is expressed as the amount of 4-MU produced in one minute by one mg of protein extracted from the cells. a, p<0.01 as compared with pBI/CSmybA1. b, p<0.05 as compared with non-transformant cells (NC). (D) RT-PCR analysis. *MybA1*-*GUS* transcripts were detected by RT-PCR analysis using MGfw and MGrv primers. CS, pBI/CSmybA1. KS, pBI/KSmybA1. Myb-GUS, *MybA1*-*GUS* mRNA. Actin, an internal control.

Gene fragments between the second exon and the third exon, including the second intron, of Cabernet Sauvignon or Koshu *MybA1* gene were inserted in front of the GUS reporter gene (Figure 5A). Also, to determine whether the 33 bp short insertion has any sequence-specific effects in the second intron, we made two deletion mutants (pBI/KS17 and pBI/KS16) and an antisense mutant (pBI/KS33R) of the 33 bp short insertion (Figure 5B). GUS activity of BY-2 cells transformed by pBI/KSmybA1 was less than one-third of BY-2 cells transformed by pBI/CSmybA1 (Figure 5C). BY-2 cells transformed by an antisense mutant pBI/KS33R showed high GUS activity. A deletion mutant pBI/KS16 decreased GUS activity similarly to pBI/KSmybA1, while GUS activity of BY-2 cells transformed by pBI/KS17 was similar to that of pBI/CSmybA1 (Figure 5C). Although *MybA1*-*GUS* mRNA was detected in both transformants by RT-PCR analysis, BY-2 cells transformed by pBI/CSmybA1 expressed the transcripts more abundantly than those transformed by pBI/KSmybA1 (Figure 5D).

Taken together, these results suggest that the 33 bp short insertion in the second intron of the Koshu *MybA1* gene decreases the amount of transcripts of the reporter gene, and that 16 bp of the 3′ end of the 33 bp short insertion is essential sequences for a defect in splicing of *MybA1* transcripts in Koshu grape, but not that the length of the short insertion.

### Prediction of RNA secondary structure of second intron of Koshu *MybA1* gene

**Figure 6:**
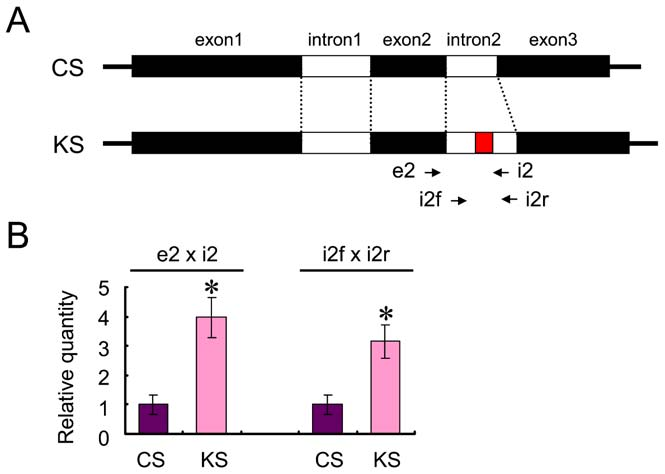
Detection of unspliced second intron in Koshu berry skin. (A) Primer map to detect unspliced second intron. (B) RT-PCR analysis using two primer sets. Total RNAs extracted from berry skin of Cabernet Sauvignon (CS) and Koshu (KS) were used. *p<0.05 as compared with CS.

The second intron of the Koshu *MybA1* gene is 152 bp, whereas that of Cabernet Sauvignon *MybA1* gene is 119 bp. To determine the effect of the 33 bp short insertion on the splicing efficiency of the second intron in the *MybA1* pre-matured transcripts in Koshu and Cabernet Sauvignon berry skins, two primer sets for intron splicing assay were used (Figure 6A). The unspliced second intron of *MybA1* in total RNA was detected by more than three to four times in Koshu skin compared with that in Cabernet Sauvignon skin (Figure 6B). Considering that Koshu berry skin expressed low levels of MybA1 at harvest compared with Cabernet Sauvignon berry skin (Figure 2), this result suggests that the 33 bp short insertion in the second intron of the Koshu *MybA1* gene may generate and accumulate frequently unspliced *MybA1* mRNA with the second intron in Koshu berry skin, but not affect transcription activity.

## Discussion

Sequence polymorphisms, including 44 bp and 111 bp insertions, in the promoter of *VvmybA1* have been reported by other groups [11]–[13]. Although the gene structure of *MybA1* is a major determinant of grape berry color [11], these insertions in the *MybA1* promoter seem to be not closely related to grape berry color. Although the 44 bp insertion is associated with red or pink grape berries in *V. vinifera* [12], other *Vitis* species, such as *V. labruscana* [13], have black berries irrespective of the 44 bp insertion. This study also demonstrated that the 44 bp and 111 bp insertions were spread to black *Vitis* species, namely, *V. labrusca*, *V. rupestris*, *V. riparia*, *V. coignetiae,* and *V. amurensis* (Figure 4). Therefore, both insertions do not function as the determinants for the color variation of grape berries, although the functional significance of the insertions in the *MybA1* promoter of *Vitis* species remains to be clarified.

Another 33 bp short insertion in the *MybA1* second intron was also discovered by two research groups [11], [13]. Lijavetzky et al. [11] found the short insertion in Sultanina (white-skinned *V. vinifera*), Sultanina Rosée (pink-skinned *V. vinifera*), and Sultanina Rouge (pink-skinned *V. vinifera*) and called it *VvmybA1^SUB^*. They analyzed the gene structure of *MybA1* in 189 grape cultivars, but did not detect the short insertion in black/red cultivars. The present study demonstrated that pink-colored oriental *V. vinifera* cultivars tested have the 33 bp short insertion in the *VvmybA1* second intron without exception. Our additional data showed that oriental *V. vinifera* cvs. Baijixin and Niunai, bearing green-yellow berries, also have this short insertion at the same position (data not shown). The gene expression of *VvmybA1^SUB^* was confirmed in the skin of pink *V. vinifera* cvs. Sultanina Rosée and Sultanina Rouge berries [11] as well as pink *V. vinifera* cvs. Koshu, Ryugan, and Huotianhong berries. Sultanina, Sultanina Rosée, and Sultanina Rouge are categorized into oriental *V. vinifera* West Asian cultivars and formed a cluster with the two Chinese cultivars, Ryugan and Huotianhong [2]. Considering that occidental *V. vinifera* cultivars, such as Cabernet Sauvignon, Pinot noir, and Merlot, never have the 33 bp short insertion in their *MybA1* second intron as well as the 44 bp and 111 bp insertions in their *MybA1* promoter, these insertions provide some information about the evolution and domestication of grapes. In fact, *V. vinifera orientalis* cultivars were genetically away from *V. vinifera occidentalis* cultivars (Figure S4). At least, the Japanese *V. vinifera* cultivar Koshu is presumed to be transported and evolved along the silk road from Turkey to Japan through China.

The 33 bp short insertion in the second intron of *MybA1* affected the splicing and maturation of the *MybA1* transcript, resulting in the low expression of *MybA1* in grape berry skin (Figures 5 and 6). The insertion, however, did not affect the transcription activity of the *MybA1* gene, because unspliced *MybA1* mRNA was accumulated in Koshu berry skin (Figure 6B). In maize, the insertion of non-intronic sequences into introns interfered with mRNA splicing, resulting in a reduction in the mRNA content [14]. Although the actual level of spliced *MybA1* transcripts was not measured in this study, unspliced *MybA1* mRNA is generated by more than three to four times in Koshu berry skin compared with that in Cabernet Sauvignon berry skin (Figure 6B). In addition, the length of the short insertion is not essential for a defect in splicing of *MybA1* transcripts. The experiments using deletion mutants of the 33 bp short insertion demonstrated that 16 bp of the 3′ end of the 33 bp short insertion is a key sequence. Consequently, unspliced *MybA1* may be degraded by an unknown mechanism, resulting in the suppression of anthocyanin synthesis due to less MybA1 protein in the skin.

Where did the 33 bp short insertion in the *MybA1* second intron come from? One possibility is that a transposable element was involved in the insertion. Both the copy number and the distribution of retrotransposons in grape genome are one of the signposts for grape evolution and domestication [5], [15], [16]. However, so far, we could not find any nucleotide sequences corresponding to the 33 bp short insertion from the grapevine genome and the EST sequences in NCBI databases and grape Massively Parallel Signature Sequencing (MPSS) database (http://mpss.udel.edu/grape/). As the databases are constructed from the sequences of occidental *V. vinifera* cultivars, further investigations are necessary to elucidate where the 33 bp short insertion came from and how it was inserted in the *MybA1* second intron.

From the above study, we hypothesize that pink grape berry is the result of the short insertion in the intron of the color regulatory gene *MybA1*. This is a new hypothesis concerning the molecular mechanism underlying the fate of grape berry skin color, although we could not demonstrate the direct evidence that unspliced *MybA1* transcripts does not function to induce anthocyanin biosynthesis in plants. Future studies to understand the function of spliced and unspliced *MybA1* transcripts isolated from pink-colored grape would prove our hypothesis.

## Methods

### Plant materials

Young leaves of *Vitis vinifera* cvs. Cabernet Sauvignon, Merlot, Syrah, Pinot Noir, Koshu, Riesling, and Chardonnay; *V. labrusca* cv. Concord; *V. labruscana* cv. Delaware; *V. riparia* cv. Gloire de Montpellier; and *V. rupestris* cv. St. George were collected from an experimental vineyard at the University of Yamanashi. Young leaves of *V. vinifera* cvs. Ryugan and Huotianhong were supplied by the National Research Institute of Brewing, Japan. Young leaves of *V. coignetiae* Pulliat, *V. ficifolia* var. Lobata Nakai, *V. saccharifera* Makino, *V. flexuosa* Thunb., *V. shiragai* Makino, and *V. amurensis* Rupr. were obtained from the University Farm of Kyoto Prefectural University, Japan.

### Measurement of anthocyanin composition

Anthocyanin (cyanidin, peonidin, delphinidin, petunidin, and malvidin) composition in berry skins was measured using reversed-phase high performance liquid chromatography as described previously [3]. Berries were collected at 19 weeks post flowering.

### RNA isolation

RNA was isolated from plant materials as described by Furiya et al. [9].

### Real-time PCR

First-strand cDNA was synthesized from total RNA using a PrimeScript RT Reagent Kit (Takara, Otsu, Japan) and real-time PCR was performed using an SYBR Premix Ex Taq II (Takara), according to the manufacturer's instructions. Nucleotide sequences of the primers used in this study were as follows: *UFGT* primers (5′-CCCTTACGTTACAGGCATTCAAG-3′ and 5′-TGCGTGAGAAGAGCGAGTTTAG-3′, corresponding to bases 554-576 and 684-663 of *V. vinifera UFGT*, GenBank accession no. AF000372, respectively), *VvmybA1* primers (5′-GCAAGCCTCAGGACAGAAGAA-3′ and 5′-ATCCCAGAAGCCCACATCAA-3′, corresponding to bases 607–627 and 720–701 of *V. vinifera VvmybA1*, GenBank accession no. AB111101 CDS region, respectively), and *β-actin* primers (5′-CAAGAGCTGGAAACTGCAAAGA-3′ and 5′-AATGAGAGATGGCTGGAAGAGG-3′, corresponding to bases 409–430 and 537–516 of *V. vinifera β-actin*, GenBank accession no. AF369524, respectively). Primers for intron splicing assay were e2 (5′-TCAAGAGAGGAGAGTTTGCATTAG-3′), i2 (5′-ATACACGCACAACATCAGACAAAG-3′), i2f (5′-GCAAGTCTATAATAACTCAAGTACT-3′), and i2r (5′-CTATACACGCACAACATCAGACAAA-3′). β-Actin was used for normalization and expression levels were expressed as relative values.

### Reverse transcription-PCR

Reverse transcription-PCR (RT-PCR) was performed according to a previously published method [9]. Nucleotide sequences of the primers used in this study were as follows: MGfw, 5′-CTGTTCAGTTGATACTTTGT-3′ and MGrv, 5′-GGCTTCAAATGGCGTATAGC-3′ (Figure 5A). PCR conditions were as follows: after incubation at 95°C for 5 min, PCR amplification was performed for 25 cycles at 95°C for 20 s, 60°C for 30 s, and 72°C for 30 s, followed by a final extension step at 72°C for 10 min. The PCR products were separated on a 2.0% agarose gel and visualized by ethidium bromide staining under ultraviolet illumination.

### DNA isolation

Genomic DNA was isolated from young leaves using a DNeasy Plant Mini Kit (Qiagen, Valencia, CA) according to the manufacture's instructions.

### Analysis of *MybA1* gene structure by PCR

PCR primers a, b, and c, designed by Kobayashi et al. [5], were used to amplify the 5′ flanking region and the coding region of the *MybA1* genes from white and red alleles (Figure 3A). Briefly, PCR was performed with 50 ng of genomic DNA using the following PCR cycling conditions: 95°C for 3 min, 35 cycles of 94°C for 30 sec, 65°C for 30 sec, and 72°C for 1.5 min, followed by a final extension step at 72°C for 10 min. The PCR products were separated on a 1.5% agarose gel and visualized by ethidium bromide staining under ultraviolet illumination. PCR products amplified with primers b and c or a and c, corresponding to the red or white alleles of the *MybA1* gene, respectively, were sequenced with a dye-terminator cycle-sequencing reaction (Greiner Japan, Tokyo, Japan). Alignment analysis of the nucleotide sequences of the *MybA1* genes was performed with ClustalW version 1.83.

Cluster analysis of the *MybA1* genes was performed using the neighbor-joining (NJ) method with bootstrap analysis using Molecular Evolutionary Genetics Analysis software (www.megasoftware.net).

### Plasmid construction for intron functional analysis

Gene fragments from the second exon to the third exon, including the second intron, were amplified from the genomic DNA of Cabernet Sauvignon and Koshu by PCR using primers 5′-GCGTCTAGA *ATG*TTGAGATGGCTCAATTATTT-3′ containing an *Xba*I site (underline) and ATG start codon (italic) and 5′-CGTGGATCCCTTGACATCATTAGCAGTCCT-3′ containing a *Bam*HI site (underline) (Figure 5A). The PCR product was digested by *Xba*I and *Bam*HI and ligated into the *Xba*I and *Bam*HI sites of binary vector pBI121 (Clontech), resulting in a pBI/CSmybA1 plasmid from Cabernet Sauvignon or a pBI/KSmybA1 plasmid from Koshu, respectively (Figure 5A).

To construct two deletion and an antisense mutants of 33 bp short insertion, inverse PCR was performed from pBI/KSmybA1 using the following primers; 5′-TTTGTAAGTTCTGAACAGCTTCAG-3′ and 5′-TTAGAAAAGCCCCCATGAGAGCTGTTCAGTTGATAC-3′ for deletion of 3′ end of the 33 bp short insertion, 5′-TTTGTAAGTTCTGAACAGCTTCAG-3′ and 5′-AATTAGAACTTACAAAAGAGCTGTTCAGTTGATAC-3′ for deletion of 5′ end of the 33 bp short insersion, or 5′-TTTGTAAGTTCTGAACAGCTTCAG-3′ and 5′-TTTGTAAGTTCTAATTCATGGGGGCTTTTCTAAAGAGCTGTTCAGTTGATAC-3′ for antisense of 33 bp short insertion. The PCR products were self-ligated, resulting in pBI/KS17, pBI/KS16, and pBI/KS33R, respectively (Figure 5B).

### Transformation

Tobacco BY-2 cells were transformed using pBI/CSmybA1 or pBI/KSmybA1 plasmids according to previously described methods [17].

### β-Glucuronidase reporter assay

GUS activity of the transformed BY-2 cells was also determined using a FluorAce β-glucuronidase reporter assay kit (Bio-Rad, Hercules, CA) according to the manufacturer's instructions. GUS activity was expressed as the amount of 4-methyl umbelliferone (4-MU) produced in one minute by one mg of protein. The experiments for GUS staining and GUS activity were carried out using three independent transformants.

### Statistics

Data are presented as means ± standard deviations. Statistical analysis was performed using the Student's *t*-test and ANOVA (analysis of variance) using Excel statistics software (Social Survey Research Information, Tokyo, Japan).

## Supporting Information

Figure S1 **Pathway leading to the synthesis of anthocyanins.** MybA1 is a transcription factor that regulates the transcription of the *UFGT* gene. PAL, phenylalanine ammonia lyase. C4H, cinnamate 4-hydroxylase. 4CL, 4-coumarate ligase. C3H, coumarate-3-hydroxylase. STS, stilbene synthase. CHS, chalcone synthase. CHI, chalcone isomerase. F3′H, flavonoid 3′-hydroxylase. F3′,5′H, flavonoid 3′,5′-hydroxylase. F3H, flavonone-3-hydroxylase. DFR, dihydroflavonol 4-reductase. LDOX, leucoanthocyanidin dioxygenase. UFGT, UDP-glucose:flavonoid 3-o-glucosyltransferase.
(PDF)

Figure S2 **Alignment of the nucleotide sequences of the** ***MybA1*** **gene between Koshu and Cabernet Sauvignon.** (A) Promoter and coding regions of *MybA1* in the red allele. (B) *MybA1* coding regions in the white allele. Koshu has three additional gene fragments (44 bp, 111 bp, and 33 bp, shaded black) in the red allele of the *MybA1* gene relative to the sequence of Cabernet Sauvignon. The DNA sequence of the *MybA1* gene of the white allele is identical between Koshu and Cabernet Sauvignon. CS, Cabernet Sauvignon. KS, Koshu.
(PDF)

Figure S3 **Alignment of nucleotide sequences of** ***MybA1*** **genes of the red allele among grapes.** The 33 bp short fragments in the second intron of the *MybA1* gene of oriental *V. vinifera* cultivars are shaded black. Identical nucleotides are indicated by asterisks. Vfl, *V. flexuosa*. KS, Koshu. Ryu, Ryugan. HU, Huotianhong. Vshi3, *V. shiragai* red allele 3. Vsa2, *V. saccharifera* red allele 2. Vshi2, *V. shiragai* red allele 2. Vfi2, *V. ficifolia* red allele 2. CS, Cabernet Sauvignon. Syr, Syrah. PN, Pinot Noir. Me, Merlot. Vco2, *V. coignetiae* red allele 2. Vamu2, *V. amurensis* red alelle 2. Vlcon, *V. labrusca* cv. Concord. Vru, *V. rupestris*. Vri, *V. riparia*. Vldela, *V. labruscana* cv. Delaware. Vco1, *V. coignetiae* red allele 1. Vamu1, *V. amurensis* red alelle 1. Vfi1, *V. ficifolia* red allele 1. Vshi1, *V. shiragai* red allele 2. Vsa1, *V. saccharifera* red allele 1.
(PDF)

Figure S4 Phylogenetic tree of ***MybA1*** genes of the red allele among grapes. Bootstrap values are indicated on the branches. CS, Cabernet Sauvignon. ME, Merlot. SYR, Syrah. PN, Pinot Noir. KS, Koshu. RYU, Ryugan. HU, Huotianhong.
(PDF)

Table S1 Size of *MybA1* PCR products amplified from red alleles in *Vitis* species.
(PDF)
