# Supplementary material for: Pink-Colored Grape Berry Is the Result of Short Insertion in Intron of Color Regulatory Gene
Source: PLoS One. 2011 Jun 17;6(6):e21308. doi: 10.1371/journal.pone.0021308 (PMC3117884; doi:10.1371/journal.pone.0021308)
Supplement: Figure S1 — Pathway leading to the synthesis of anthocyanins. MybA1 is a transcription factor that regulates the transcription of the UFGT gene. PAL, phenylalanine ammonia lyase. C4H, cinnamate 4-hydroxylase. 4CL, 4-coumarate ligase. C3H, coumarate-3-hydroxylase. STS, stilbene synthase. CHS, chalcone synthase. CHI, chalcone isomerase. F3′H, flavonoid 3′-hydroxylase. F3′,5′H, flavonoid 3′,5′-hydroxylase. F3H, flavonone-3-hydroxylase. DFR, dihydroflavonol 4-reductase. LDOX, leucoanthocyanidin dioxygenase. UFGT, UDP-glucose:flavonoid 3-o-glucosyltransferase. (PDF) [file pone.0021308.s001.pdf]

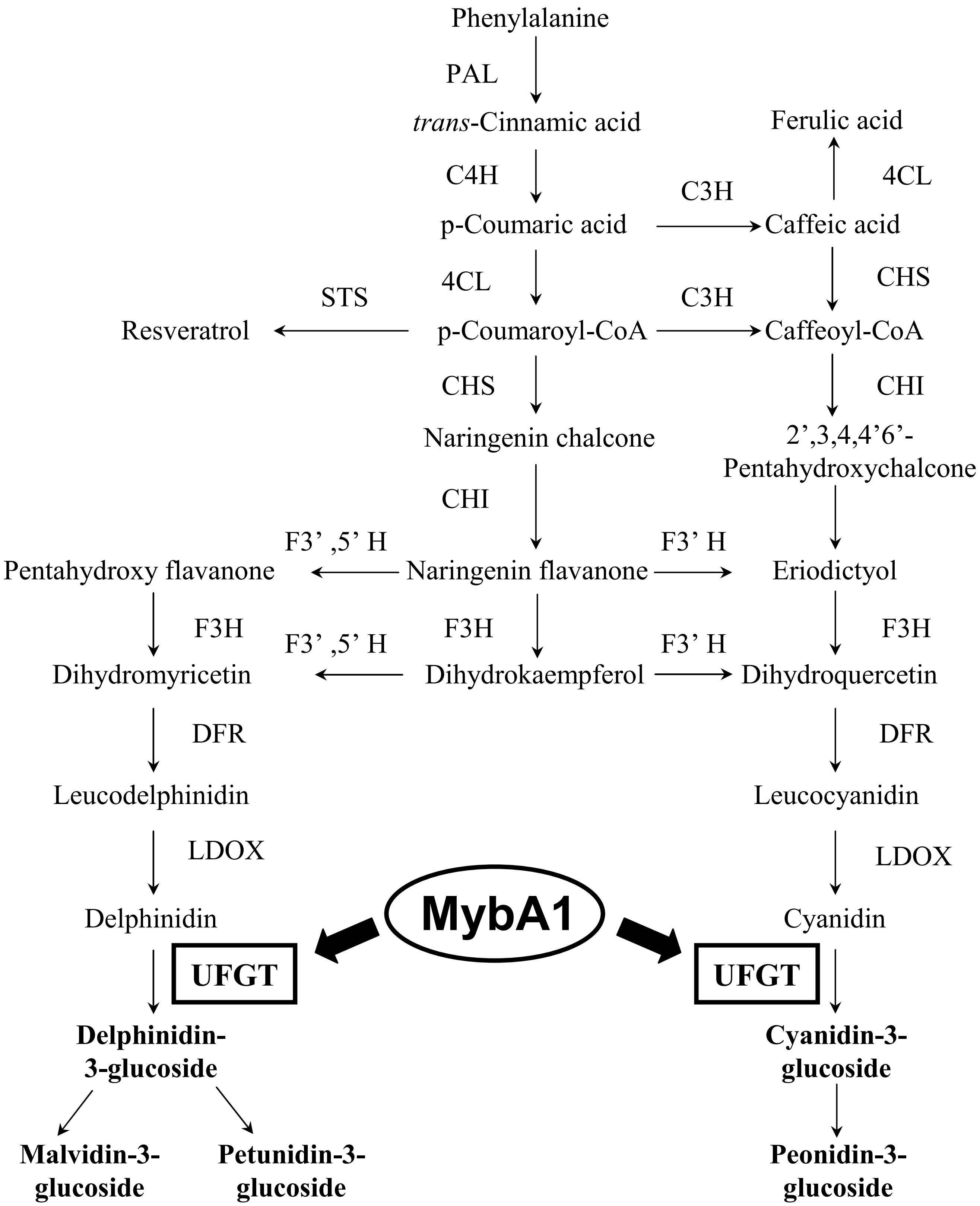

Figure S1. Pathway leading to the synthesis of anthocyanins. MybA1 is a transcription factor that regulates the transcription of the *UFGT* gene. PAL, phenylalanine ammonia lyase. C4H, cinnamate 4-hydroxylase. 4CL, 4-coumarate ligase. C3H, coumarate-3-hydroxylase. STS, stilbene synthase. CHS, chalcone synthase. CHI, chalcone isomerase. F3'H, flavonoid 3'-hydroxylase. F3',5'H, flavonoid 3',5'-hydroxylase. F3H, flavonone-3-hydroxylase. DFR, dihydroflavonol 4-reductase. LDOX, leucoanthocyanidin dioxygenase. UFGT, UDP-glucose:flavonoid 3-o-glucosyltransferase.
