# Supplementary material for: Pink-Colored Grape Berry Is the Result of Short Insertion in Intron of Color Regulatory Gene
Source: PLoS One. 2011 Jun 17;6(6):e21308. doi: 10.1371/journal.pone.0021308 (PMC3117884; doi:10.1371/journal.pone.0021308)
Supplement: Figure S2 — Alignment of the nucleotide sequences of the MybA1 gene between Koshu and Cabernet Sauvignon. (A) Promoter and coding regions of MybA1 in the red allele. (B) MybA1 coding regions in the white allele. Koshu has three additional gene fragments (44 bp, 111 bp, and 33 bp, shaded black) in the red allele of the MybA1 gene relative to the sequence of Cabernet Sauvignon. The DNA sequence of the MybA1 gene of the white allele is identical between Koshu and Cabernet Sauvignon. CS, Cabernet Sauvignon. KS, Koshu. (PDF) [file pone.0021308.s002.pdf]

A

```
CS   GGACGTTAAAAAATGGTTGCACGTGGTTGTCTTCAGGATCACACCAGTTTATACATTTGGACCACAAAATAGAGATTGTTCATCAAGGAT
KS   GGACGTTAAAAAATGGTTGCACGTGGTTGTCTTCAGGATCACACCAGTTTCTACATTTGGACCACAAAATAGAGATCGTTCATAAAGGAT

                                  44 bp insertion
CS   ACTAGTCAGCAATTAATTCCTAAAT--------------------------------------------ATCTCTTATGACACACACCCT
KS   ACTAGTCAGCAATTAATTCCTAAATTTTGCGCTGTACATTTATAGTAAGTTGATACATAATGGGTAAATATCTCTTATGACACACACCCT

CS   TTGTCCATGAACTCCAGCGCATTTGGAAGCCAG-TAATGCACCATAAGAAACGTGTCGAATAAACCAATTAGGGGTCTGGTGTCCGAGTC
KS   TTGTCCACGAACTCCAGCGCATTCGGAAGCCAGGTAATGCACCATAAGAAACGGGTCGAATAAACCAATTAGGGGTCTGGTGTCCGAGTC

                                                        114 bp insertion
CS   ATGAGATAGAACAGGTTCGAGGTT-----------------------------------------------------------------
KS   ATGAGATAGAACAGGTTCGAGGTTATTTTATGGATTTTTCTTTTTACCAAAAAGAAAAAGCATCCGGTTATAGGAAAAGGAAAAATATC

CS   ---------------------------------------------GTTATATATCAATCAATAATTAGAGAAGGAGCCGGTCTCTTGTGT
KS   CCAAGGAACATAGTAAACAAAGATAGGAAATACAGTTTTGTGGTTGTTATATATCAATCAATAATTAGAGAAGGAGCTGGTCTCTTGTGT

CS   TGAGTTGACTCGATGGAGAGCTTAGGAGTTAGAAAGGGTGCATGGATCCAAGAAGAGGATGTTCTCCTGAGGAAATGCATTGAGAAATAT
KS   TGAGTTGACTCTATGGAGAGCTTAGGAGTTAGAAAGGGTGCATGGATCCAAGAAGAGGATGTTCTCCTGAGGAAATGCATTGAGAAATAT

CS   GGAGAAGGAAAGTGGCATCTGGTTCCCCTCCGAGCAGGTAACATGAAAGAGAAAGGGATCAGTATTTATTTGTGTTTTTTTACTTCTGTT
KS   GGAGAAGGAAAGTGGCATCTGGTTCCCCTCCGAGCAGGTAACATGAAAGAGAAAGGGATCAGTATTAATTTGTGTTTTATTACTTCTGTT

CS   TTGCTTAAAGAGTTTCATTTTCTTGAGTTTGCAGGGTTGAATAGATGCCGAAAAAGCTGCAGGTTGAGATGGCTCAATTATTTGAAGCCG
KS   TTGCTTAAAGAGTTTCGTTTTCTTGAGTTTGCAGGGTTGAATAGATGCCGAAAAAGCTGCAGATTGAGATGGCTCAATTATTTGAAGCCG

CS   GATATCAAGAGAGGAGAGTTTGCATTAGACGAGGTTGATCTCATGATTAGGCTTCACAATTTGTTGGGGAACAGGCAAGTCTATAATAAC
KS   GATATCAAGAGAGGAGAGTTTGCATTAGACGAGGTTGACCTCATGATTAGGCTTCACAATTTGTTGGGGAACAGGCAAGTCTATAATAAC

                                                                          33 bp insertion
CS   TCAAGTACTAGCTTGATAATGATATTATATTAGTTCTGAAGCTGTTCAGAACTTACAAA-------------------------------
KS   TCAAGTACTAGCTTGATAATGATATTATATTAGTTCTGAAGCTGTTCAGAACTTACAAATTAGAAAAGCCCCCATGAATTAGAACTTACA

CS   --AGAGCTGTTCAGTTGATACTTTGTCTGATGTTGTGCGTGTATAGATGGTCCTTGATTGCGGGTAGGCTTCCAGGGAGGACTGCTAATG
KS   AAAGAGCTGTTCAGTTGATACTTTGTCTGATGTTGTGCGTGTATAGATGGTCCTTGATTGCGGGTAGGCTTCCAGGGAGGACTGCTAATG

CS   ATGTCAAGAACTATTGGCATAGTCACCACTTCAAAAAGGAGGTTC
KS   ATGTCAAGAACTATTGGCATAGTCACCACTTCAAAAAGGAGGTTC
```

B

```
CS   ATGGAGAGCTTAGGAGTTAGAAAGGGTGCATGGATCCAAGAAGAGGATGTTCTCCTGAGGAAATGCATTGAGAAATATGGAGAAGGAAAG
KS   ATGGAGAGCTTAGGAGTTAGAAAGGGTGCATGGATCCAAGAAGAGGATGTTCTCCTGAGGAAATGCATTGAGAAATATGGAGAAGGAAAG

CS   TGGCATCTGGTTCCCCTCCGAGCAGGTAACATGAAAGAGAAAGGGATCAGTATTTATTTGTGTTTTTTTACTTCTGTTTTGCTTAAAGAG
KS   TGGCATCTGGTTCCCCTCCGAGCAGGTAACATGAAAGAGAAAGGGATCAGTATTTATTTGTGTTTTTTTACTTCTGTTTTGCTTAAAGAG

CS   TTTCATTTTCTTGAGTTTGCAGGGTTGAATAGATGCCGAAAAAGCTGCAGGTTGAGATGGCTCAATTATTTGAAGCCGGATATCAAGAGA
KS   TTTCATTTTCTTGAGTTTGCAGGGTTGAATAGATGCCGAAAAAGCTGCAGGTTGAGATGGCTCAATTATTTGAAGCCGGATATCAAGAGA

CS   GGAGAGTTTGCATTAGACGAGGTTGATCTCATGATTAGGCTTCACAATTTGTTGGGGAACAGGCAAGTCTATAATAACTCAAGTACTAGC
KS   GGAGAGTTTGCATTAGACGAGGTTGATCTCATGATTAGGCTTCACAATTTGTTGGGGAACAGGCAAGTCTATAATAACTCAAGTACTAGC

CS   TTGATAATGATATTATATTAGTTCTGAAGCTGTTCAGAACTTACAAAAGAGCTGTTCAGTTGATACTTTGTCTGATGTTGTGCGTGTATA
KS   TTGATAATGATATTATATTAGTTCTGAAGCTGTTCAGAACTTACAAAAGAGCTGTTCAGTTGATACTTTGTCTGATGTTGTGCGTGTATA

CS   GATGGTCCTTGATTGCGGGTAGGCTTCCAGGGAGGACTGCTAATGATGTCAAGAACTATTGGCATAGTCACCACTTCAAAAAGGAGGTTC
KS   GATGGTCCTTGATTGCGGGTAGGCTTCCAGGGAGGACTGCTAATGATGTCAAGAACTATTGGCATAGTCACCACTTCAAAAAGGAGGTTC
```

Figure S2. Alignment of the nucleotide sequences of the *MybA1* gene between Koshu and Cabernet Sauvignon. (A) Promoter and coding regions of *MybA1* in the red allele. (B) *MybA1* coding regions in the white allele. Koshu has three additional gene fragments (44 bp, 111 bp, and 33 bp, shaded black) in the red allele of the *MybA1* gene relative to the sequence of Cabernet Sauvignon. The DNA sequence of the *MybA1* gene of the white allele is identical between Koshu and Cabernet Sauvignon. CS, Cabernet Sauvignon. KS, Koshu.
