# Supplementary material for: Pink-Colored Grape Berry Is the Result of Short Insertion in Intron of Color Regulatory Gene
Source: PLoS One. 2011 Jun 17;6(6):e21308. doi: 10.1371/journal.pone.0021308 (PMC3117884; doi:10.1371/journal.pone.0021308)
Supplement: Figure S3 — Alignment of nucleotide sequences of MybA1 genes of the red allele among grapes. The 33 bp short fragments in the second intron of the MybA1 gene of oriental V. vinifera cultivars are shaded black. Identical nucleotides are indicated by asterisks. Vfl, V. flexuosa. KS, Koshu. Ryu, Ryugan. HU, Huotianhong. Vshi3, V. shiragai red allele 3. Vsa2, V. saccharifera red allele 2. Vshi2, V. shiragai red allele 2. Vfi2, V. ficifolia red allele 2. CS, Cabernet Sauvignon. Syr, Syrah. PN, Pinot Noir. Me, Merlot. Vco2, V. coignetiae red allele 2. Vamu2, V. amurensis red alelle 2. Vlcon, V. labrusca cv. Concord. Vru, V. rupestris. Vri, V. riparia. Vldela, V. labruscana cv. Delaware. Vco1, V. coignetiae red allele 1. Vamu1, V. amurensis red alelle 1. Vfi1, V. ficifolia red allele 1. Vshi1, V. shiragai red allele 2. Vsa1, V. saccharifera red allele 1. (PDF) [file pone.0021308.s003.pdf]

```
Vfl             GGACGTTAAAAAATGGTTGCACGTGGTTGTCTTCAGGATCACACCAGTTTATACATTTGG
KS              GGACGTTAAAAAATGGTTGCACGTGGTTGTCTTCAGGATCACACCAGTTTCTACATTTGG
Ryu             GGACGTTAAAAAATGGTTGCACGTGGTTGTCTTCAGGATCACACCAGTTTCTACATTTGG
HU              GGACGTTAAAAAATGGTTGCACGTGGTTGTCTTCAGGATCACACCAGTTTCTACATTTGG
Vshi3           GGACGTTAAAAAATGGTTGCACGTGGTTGTCGTCAGGATCACACCAGTTTATACATTTGG
Vsa2            GGACGTTAAAAAATGGTTGCACGTGGTTGTCTTCAGGATCACACCAGTTTATACATTTGG
Vshi2           GGACGTTAAAAAATGGTTGCACGTGGTTGTCGTCAGGATCACACCAGTTTATACACTTGG
Vfi2            GGACGTTAAAAAATGGTTGCACGTGGTTGTCGTCAGGATCACACCAGTTTATACATTTGG
CS              GGACGTTAAAAAATGGTTGCACGTGGTTGTCTTCAGGATCACACCAGTTTATACATTTTG
Syr             GGACGTTAAAAAATGGTTGCACGTGGTTGTCTTCAGGATCACACCAGTTTATACATTTGG
PN              GGACGTTAAAAAATGGTTGCACGTGGTTGTCTTCAGGATCACACCAGTTTATACATTTGG
Me              GGACGTTAAAAAATGGTTGCACGTGGTTGTCTTCAGGATCACACCAGTTTATACATTTGG
Vco2            GGACGTTAAAAAATGGTTGCACGTGGTTGTCTTCAGGATCACACCAGTTTATACATTTGG
Vamu2           GGACGTTAAAAAATGGTTGCACGTGGTTGTCTTCAGGATCACACCAGTTTATACATTTGG
Vlcon           GGACGTTAAAAAATGGTTGCACGTGGTTGTCTTCAGGATCACACCAGGTTATACATTTGG
Vru             GGACGTTAAAAAATGGTTGCACGTGGTTGTCTTCAGGATCACACCAGG------------
Vri             GGACGTTAAAAAATGGTTGCACGTGGTTGTCTTCAGGATCACACCAGG------------
Vldela          GGACGTTAAAAAATGGTTGCACGTGGTTGTCTTCAGGATCACACCAGGTTATACATTTGG
Vco1            GGACGTTAAAAAATGGTTGCACGTGGTTGTCTTCAGGATCACACCAGGTTATACATTTGG
Vamu1           GGACGTTAAAAAATG-TTGCACGTGGTTGTCTTCAGGATCACACCAGGTTATACATTTGG
Vfi1            GGACGTTAAAAAATGGTTGCACGTGGTTGTCTTCAGGATCACACCAGTTTATACATTTGG
Vshi1           GGACGTTAAAAAATGGTTGCACGTGGTTGTCTTCAGGATCACACCAGTTTATCCATTTGG
Vsa1            GGACGTTAAAAAATGGTTGCACGTGGTTGTCTTCAGGATCACACCAGTTTATACATTTGG
                *************** *************** ***************


Vfl             ACCACAAAATAGAGATTGTTCATAAAGGATACTAGTCAGCAATTAATTCCTAAATTTTGT
KS              ACCACAAAATAGAGATCGTTCATAAAGGATACTAGTCAGCAATTAATTCCTAAATTTTGC
Ryu             ACCACAAAATAGAGATCGTTCATAAAGGATACTAGTCAGCAATTAATTCCTAAATTTTGC
HU              ACCACAAAATAGAGATCGTTCATAAAGGATACTAGTCAGCAATTAATTCCTAAATTTTGC
Vshi3           ACCACAAAATAGAGATTGTTCATAAAGGATACTAGTCAGCAATTAATTCCTAAATTTTGC
Vsa2            ACCGCAAAATAGAGATTGTTCATAAAGGATACTAGTCAGAAATTAATTCCTAAATTTTGC
Vshi2           ACCACAAAATAGAGATTGTTCATAAAGGATACTAGTCAGCAATTAATTCCTAAATTTTGC
Vfi2            ACCACAAAATAGAGATTGTTCATAAAGGATACTAGTCAGCAATTAATTCCTAAATTTTGC
CS              ACCACAAAATAGAGATTGTTCATCAAGGATACTAGTCAGCAATTAATTCCTAAAT-----
Syr             ACCACAAAATAGAGATTGTTCATCAAGGATACTAGTCAGCAATTAATTCCTAAAT-----
PN              ACCACAAAATAGAGATTGTTCATCAAGGATACTAGTCAGCAATTAATTCCTAAAT-----
Me              ACCACAAAATAGAGATTGTTCATCATGAATACTAGTCAGCAATTAATTCCTAAAT-----
Vco2            ACCACAAAATACAGATTGTTCATAAAGGATACTAGTCAGCAATTAATTCCTAAAT-----
Vamu2           ACCACAAAATACAGATTGTTCATAAAGGATACTAGTCAGCAATTAATTCCTAAAT-----
Vlcon           ACCACAAAATAGAGATTGTTCATAAAGGATACTAGTCAGCAATTAATTCCTAAATTTTGC
Vru             ACCACAAAATAGAGATTGTTCATAAAGGATACTAGTCAGCAATTAATTCCTAAATTTTGC
Vri             ACCACAAAATAGAGATTGTTCATAAAGGATACTAGTCAGCAATTAATTCCTAAATTTTGC
Vldela          ACCACAAAATAGAGATTGTTCATATAGGATACTAGTCAGCAATTAATTCCTAAATTTTGC
Vco1            ACCACAAAATAGAGATTGTTCATAAAGGATACTAGTCAGCAATTAATTCCTAAATTTTGC
Vamu1           ACCACAAAATAGAGATTGTTCATAAAGGATACTAGTCAGCAATTAATTCCTAAATTTTGC
Vfi1            ACCACAAAATAGAGATTGTTCATAAAGGATACTAGTCAGCAATTAATTCCTAAATTTTGC
Vshi1           ACCACAAAATAGAGATTGTTCATAAAGGATACTAGTCAGCAATTAATTCCTAAATTTTGC
Vsa1            ACCACAAAATAGAGATTGTTCATAAAGGATACTAGTCAGCAATTAATTCCTAAATTTTGC
                *** ******* **** ******   * *********** ***************
```

```
Vfl        GCTGTACATTTATAGTAAGTTGATACATAATGGGTAAATATCTCTTCTGACATACACCCT
KS         GCTGTACATTTATAGTAAGTTGATACATAATGGGTAAATATCTCTTATGACACACACCCT
Ryu        GCTGTACATTTATAGTAAGTTGATACATAATGGGTAAATATCTCTTATGACACACACCCT
HU         GCTGTACATTTATAGTAAGTTGATACATAATGGGTAAATATCTCTTATGACACACACCCT
Vshi3      ACTGTACATTTATAGTAAGTTGATACATAATGGGTAAATATCTCTTATGACACACACCCT
Vsa2       GCTGTATGTTTATAGTAAGTTGATACATAATGGGTAAATATCTCTTATGACACACACCCT
Vshi2      GCTGTACATTTATAGTAAGTTGATACATAATGGGTAAATATCTCTTATGACACACACCCT
Vfi2       GCTGTACATTTATAGTAAGTTGATACATAATGGGTAAATATCTCTTATGACATACACCCT
CS         ---------------------------------------ATCTCTTATGACACACACCCT
Syr        ---------------------------------------ATCTCTTATGACACACACCCT
PN         ---------------------------------------ATCTCTTATGACACACACCCT
Me         ---------------------------------------ATCTCTTATGACACACACCCT
Vco2       ---------------------------------------ATCTCTTATGACACACACCCT
Vamu2      ---------------------------------------ATCTCTTATGACACACACCCT
Vlcon      ACTGTACATTTATAGTAAGTTGATACATAATGGGTAAATATCTCTTATGACACACACCCT
Vru        ACTGTACATTTATAGTAAGTTGATACATAATGGGTAAATATCTCTTATGACATACACCCT
Vri        ACTGTACATTTATAGTAAGTTGATACATAATGGGTAAATATCTCTTATGACATACACCCT
Vldela     ACTGTACATTTATAGTAAGTTGATACATAATGGGTAAATATCTCTTATGACATACACCCT
Vco1       ACTGTACATTTATAGTAAGTTGATACATAATGGGTAAATATCTCTTATGACATGCACCCT
Vamu1      ACTGTACATTTATAGTAAGTTGATACATAATGGGTAAATATCTCTTATGACATGCACCCT
Vfi1       GCTGTATATTTATAGTAA-------------------ATATCTCTTATGACATACACCCT
Vshi1      GCTGTATATTTATAGTAA-------------------ATATCTCTTATGACATACACCCT
Vsa1       GCTGTACATTTATAGTAAGTTGATACATAATGGGTAAATATCTCTTATGACATACACCCT
                                                  ******* *****  ******


Vfl        TTGTCCATGAACTCCAGCGCATTTGGAAGCCAGGTAATGCACCATAAGAAACGTGTCGAA
KS         TTGTCCACGAACTCCAGCGCATTCGGAAGCCAGGTAATGCACCATAAGAAACGGGTCGAA
Ryu        TTGTCCACGAACTCCAGCGCATTCGGAAGCCAGGTAATGCACCATAAGAAACGGGTCGAA
HU         TTGTCCACGAACTCCAGCGCATTCGGAAGCCAGGTAATGCACCATAAGAAACGGGTCGAA
Vshi3      TTGTCCATGATCCCCAGCGCATTCGGAAGCCAGGTAATGCACCATAAGAAACGTGTCGAA
Vsa2       TTGTCCATGATCCCCAGCGCATTCGGAAGCCAGGTAATGCACCATAAGAA--GTGTCGAA
Vshi2      TTGTCCATGATCTCCAGCGCATTCGGAAGCCAGGTAATGCACCATAAGAA-CGTGTCGAA
Vfi2       TTGTCCATGAACTCCAGCGCATTCGGAAGCCAGGTAATGCACCATAAGAAACGTGTCGAA
CS         TTGTCCATGAACTCCAGCGCATTTGGAAGCCAG-TAATGCACCATAAGAAACGTGTCGAA
Syr        TTGTCCATGAACTCCAGCGCATTTGGAAGCCAG-TAATGCACCATAAGAAACGTGTCGAA
PN         TTGTCCATGAACTCCAGCGCATTTGGAAGCCAG-TAATGCACCATAAGAAACGTGTCGAA
Me         TTGTCCATGAACTCCAGCGCATTTGGAAGCCAG-TAATGCACCATAAGAAACGTGTCGAA
Vco2       TTGTCCATGAACTCCAGCGCATTTGGAAGCCAGGTAATGCACCATAAGAAACGTGTCGAA
Vamu2      TTGTCCATGAACTCCAGCGCATTTGGAAGCCAGGTAATGCACCATAAGAAACGTGTCGAA
Vlcon      TTGTCCATGAACTCCAGCGCATTTGGAAGCCAGGTAATGCACCATAAGAAACGTGTCGAA
Vru        TTGTCCATGAACTCCAGCGCATTTGGAAGCCAGGTAATGCACCATAAGAAACGTGTCGAA
Vri        TTGTCCATGAACTCCAGCGCATTTGGAAGCCAGGTAATGCACCATAAGAAACGTGTCGAA
Vldela     TTGTCCATGAACTCCAGCGCATTCGGAAGCCAGGTAATGCACCATAAGAAACGTGTCGAA
Vco1       TTGTCCATGAACTCCAGCGCATTTGGAAGCCAGGCAATGCACCATAAGAAACGTGTCGAA
Vamu1      TTGTCCATGAACTCCAGCGCATTTGGAAGCCAGGCAATGCACCATAAGAAACGTGTCGAA
Vfi1       TTGTCCATGAACTCCAGCGCATTTGGAAGCCAGGTAATGCACCATAAGAAACATGTCGAA
Vshi1      TTGTCCATGAACTCCAGCGCATTTGGAAGCCAGGTAATGCACCATAAGAAACATGTCGAA
Vsa1       TTGTCCATGAACTCCAGCGCATTTGGAAGCCAGGTAATGCACCATAAGAAACGTGTCGAA
           ******* ** * ********** *********  ***************    ******
```

```
Vfl             TAAACCAATTAGGGGTCTGGTGTCCGAGTCATGAGATAGAACAGGTTCGAGGTTATTTTA
KS              TAAACCAATTAGGGGTCTGGTGTCCGAGTCATGAGATAGAACAGGTTCGAGGTTATTTTA
Ryu             TAAACCAATTAGGGGTCTGGTGTCCGAGTCATGAGATAGAACAGGTTCGAGGTTATTTTA
HU              TAAACCAATTAGGGGTCTGGTGTCCGAGTCATGAGATAGAACAGGTTCGAGGTTATTTTA
Vshi3           TCAACCAATTAGGGGTCTGGTGTCCGAGTCATGAGATAGAACAGGTTCGAGGTT------
Vsa2            TCAACCAATTAGGGGTCTGGTGTCCGAGTCATGAGATAGAACAGGTTCGAGGTT------
Vshi2           TCAACCAATTAGGGGTCTGGTGTCCGAGTCATGAGATAGAACAGGTTCGAGGTT------
Vfi2            TCAACCAATTAGGGGTCTGGTGTCCGAGTCATGAGATAGAACAGGTTCGAGGTT------
CS              TAAACCAATTAGGGGTCTGGTGTCCGAGTCATGAGATAGAACAGGTTCGAGGTT------
Syr             TAAACCAATTAGGGGTCTGGTGTCCGAGTCATGAGATAGAACAGGTTCGAGGTT------
PN              TAAACCAATTAGGGGTCTGGTGTCCGAGTCATGAGATAGAACAGGTTCGAGGTT------
Me              TAAACCAATTAGGGGTCTGGTGTCCGAGTCATGAGATAGAACAGGTTCGAGGTT------
Vco2            TAAACCAATTAGGGGTCTGGTGTCCGAGTCATGAGATAGAACAGGTTCGAGGTT------
Vamu2           TAAACCAATTAGGGGTCTGGTGTCCGAGTCATGAGATAGAACAGGTTCGAGGTT------
Vlcon           TAAACCAATTAGGGGTCTGGTGTCCGAGTCATGAGATAGAACAGGTTCGAGGTTATTTTA
Vru             TAAACCAATTAGGGGTCTGGTGTCCGAG----------------------GGTTATTTTA
Vri             TAAACCAATTAGGGGTCTGGTGTCCGAG----------------------G-TTATTTTA
Vldela          TAAACCAATTAGGGGTCTGGTGTCCGAGTCATGAGATAGAACAGGTTCGAGGTTATTTTA
Vco1            TAAACCAATTAGGGGTCTGATGTCTGAGTCATGAGATAGAACAGGTTCGAGGTTATTTTA
Vamu1           TAAACCAATTAGGGGTCTGATGTCTGAGTCATGAGATAGAACAGGTTCGAGGTTATTTTA
Vfi1            TAAACCAATTAGGGGTCTGGTGTCCGAGTCATGAGATAGAACAGGTTCGAGGTTATTTTA
Vshi1           TAAACCAATTAGGGGTCTGGTGTCCGAGTCATGAGATAGAACAGGTTCGAGGTTATTTTA
Vsa1            TAAACCAATTAGGGGTCTGGTGTCCGAGTCATGAGATAGAACAGGTTCGAGGTTATTTTA
                * ***************** **** ***                      * **

Vfl             TTGATTTTTTTTTTTTACAAAAAGGAAAAAGCATCCGGTTATAGGAAAAGGAAAAATATC
KS              TGGATTTTTTCTTTTTACCAAAAAGAAAAAGCATCCGGTTATAGGAAAAGGAAAAATATC
Ryu             TGGATTTTTTCTTTTTACCAAAAAGAAAAAGCATCCGGTTATAGGAAAAGGAAAAATATC
HU              TGGATTTTTTCTTTTTACCAAAAAGAAAAAGCATCCGGTTATAGGAAAAGGAAAAATATC
Vshi3           ------------------------------------------------------------
Vsa2            ------------------------------------------------------------
Vshi2           ------------------------------------------------------------
Vfi2            ------------------------------------------------------------
CS              ------------------------------------------------------------
Syr             ------------------------------------------------------------
PN              ------------------------------------------------------------
Me              ------------------------------------------------------------
Vco2            ------------------------------------------------------------
Vamu2           ------------------------------------------------------------
Vlcon           TTGATTTTTTCTTTTTACAAAAAAGAAAAAGCATCCGGTTATAGGAAAAGGAAAAATATC
Vru             TTGATTTTTTCTTTTTACAAAAAAGAAAAAGCATCCGGTTATAGGAAAAGGAAAAATATC
Vri             TTGATTTTTTCTTTTTACAAAAAAGAAAAAGCATCCGGTTATAGGAAAAGGAAAAATATC
Vldela          TTGATTTTTTCTTTTTACAAAAAAGAAAAAGCATCCGGTTATAGGAAAAGGAAAAATATC
Vco1            TTGATTTTTTCTTTTTACAAAAAAGAAAAAGCATCCGGTTATAGGAAAAGGAAAAATATC
Vamu1           TTGATTTTTTCTTTTTACAAAAAAGAAAAAGCATCCGGATATAGGAAAAGGAAAAATATC
Vfi1            TTGATTTTTTTTTTTTACAAAAAAGAAAAAGCATCCGGTTATAGGAAAAGGAAAAATATC
Vshi1           TTGATTTTTTTTTTTTACAAAAAAGAAAAAGCATCCGGTTATAGGAAAAGGAAAAATATC
Vsa1            TTGATTTTTTTTTTT-ACAAAAAGGAAAACGCATCCGGTTATAGGAAAAGGAAAAATATC
```

```
Vfl       CCAAGGAACATAGTAAACAAAGATAGGAAGTACGGTTTTGTGGCTGTTATATATCAATCA
KS        CCAAGGAACATAGTAAACAAAGATAGGAAATACAGTTTTGTGGTTGTTATATATCAATCA
Ryu       CCAAGGAACATAGTAAACAAAGATAGGAAATACAGTTTTGTGGTTGTTATATATCAATCA
HU        CCAAGGAACATAGTAAACAAAGATAGGAAATACAGTTTTGTGGTTGTTATATATCAATCA
Vshi3     ---------------------------------------------GTTATATATCAATCA
Vsa2      ---------------------------------------------GTTATATATCAATCA
Vshi2     ---------------------------------------------GTTATATATCAATCA
Vfi2      ---------------------------------------------GTTATATATCAATCA
CS        ---------------------------------------------GTTATATATCAATCA
Syr       ---------------------------------------------GTTATATATCAATCA
PN        ---------------------------------------------GTTATATATCAATCA
Me        ---------------------------------------------GTTATATATCAATCA
Vco2      ---------------------------------------------GTTATATATCAATCA
Vamu2     ---------------------------------------------GTTATATATCAATCA
Vlcon     CCAAGGAACATAGTAAACAAAGATAGGAAGTACGGTTTTGTGGTTGTTATATATCAATCA
Vru       CCAAGGAACATAGTAAACAAAGATAGGGAGTACGGTTTTGTGGTTGTTATATATCAATCA
Vri       CCAAGGAACATAGTAAACAAAGATAGGAAGTACGGTTTTGTGGTTGTTATATATCAATCA
Vldela    CCAAGGAACATAGTAAACAAAGATAGGAAGTACGGTTTTGTGGTTGTTATATATCAATCA
Vco1      CCAAGGAACATAGTAAACAAAGATAGGAAGTACGGTTTTGTGGTTGTTATATATCAATCG
Vamu1     CCAAGGAACATAGTAAACAAAGATAGGAAGTACGGTTTTGTGGTTGTTATATATCAATCG
Vfi1      CCAAGGAACATAGTAAACAAAGATAGGAAGTACGGTTTTGTGGTTGTTATATATCAATCA
Vshi1     CCAAGGAACATAGTAAACAAAGATAGGAAGTACGGTTTTGTGGTTGTTATATATCAATCA
Vsa1      CCAAGGAACATAGTAAACAAAGATAGGAAGTACGGTTTTGTGGTTGTTATATATCAATCA
                                                         **************

Vfl       ATAATTAGAGAAGGAGCTGGTCTCTTGTGTTGAGTTGACTCCATGGAGAGCTTAGGAGTT
KS        ATAATTAGAGAAGGAGCTGGTCTCTTGTGTTGAGTTGACTCTATGGAGAGCTTAGGAGTT
Ryu       ATAATTAGAGAAGGAGCTGGTCTCTTGTGTTGAGTTGACTCTATGGAGAGCTTAGGAGTT
HU        ATAATTAGAGAAGGAGCTGGTCTCTTGTGTTGAGTTGACTCTATGGAGAGCTTAGGAGTT
Vshi3     ATAATTAGAGAAGGAGCCGGTCTCTTGTGTTGAGTTGACTCGATGGAGAGCTTAGGAGTT
Vsa2      ATAATTAGAGAAGGAGCCGGTCTCTTGTGTTGAGTTGACTCGATGGAGAGCTTAGGAGTT
Vshi2     ATACTTAGAGAAGGAGCCGGTCTCTTGTGTTGAGTTGACTCGATGGAGAGCTTAGGAGTT
Vfi2      ATAATTAGAGAAGGAGCCGGTCTCTTGTGTTGAGTTGACTCGATGGAGAGCTTAGGAGTT
CS        ATAATTAGAGAAGGAGCCGGT-TCTTGTGTTGAGTTGACTCGATGGAGAGCTTAGGAGTT
Syr       ATAATTAGAGAAGGAGCCGGTCTCTTGTGTTGAGTTGACTCGATGGAGAGCTTAGGAGTT
PN        ATAATTAGAGAAGGAGCCGGTCTCTTGTGTTGAGTTGACTCGATGGAGAGCTTAGGAGTT
Me        ATAATTAGAGAAGGAGCCGGTCTCTTGTGTTGAGTTGACTCGATGGAGAGCTTAGGAGTT
Vco2      ATAATTAGAGAAGGAGCCGGTCTCTTGTGTTGAGTTGACTCCATGGAGAGCTTAGGAGTT
Vamu2     ATAATTAGAGATGGAGCCGGTCTCTTGTGTTGAGTTGACTCCATGGAGAGCTTAGGAGTT
Vlcon     ATAATTAGAGAAGGAGCTGGTCTCTTGTGTTGAGTTGACTCCATGGAGAGCTTAGGAGTT
Vru       ATAATTAGAGAAGGAGCTGGTCTCTTGTGTTGAGTTGACTCCATGGAGAGCTTAGGAGTT
Vri       ATAATTAGAGAAGGAGCTGGTCTCTTGTGTTGAGTTGACTCCATGGAGAGCTTAGGAGTT
Vldela    ATAATTAGAGAAGGAGCTGGTCTCTTGTGTTGAGTTGACTCCATGGAGAGCTTAGGAGTT
Vco1      ATAATTAGAGAAGGAGCTGGTCTCTTGTGTTGAGTTGACTCCATGGAGAGCTTAGGAGTT
Vamu1     ATAATTAGAGAAGGAGCTGGTCTCTTGTGTTGAGTTGACTCCATGGAGAGCTTAGGAGTT
Vfi1      ATAATTAGAGAAGGAGCTGGTCTCTTGTGTTGAGTTGACTCCATGGAGAGCTTAGGAGTT
Vshi1     ATAATTAGAGAAGGAGCTGGTCTCTTGTGTTGAGTTGACTCCATGGAGAGCTTAGGAGTT
Vsa1      ATAATTAGAGAAGGAGCTGGTCTCTTGTGTTGAGTTGACTCCATGGAGAGCTTAGGAGTT
          *** ******* ***** *** ****************** *******************
```

```
Vfl       AGAAAGGGTACATGGATCCAAGAAGAGGATGTTCTCCTGAGGAAATGCATTGACAAATAT
KS        AGAAAGGGTGCATGGATCCAAGAAGAGGATGTTCTCCTGAGGAAATGCATTGAGAAATAT
Ryu       AGAAAGGGTGCATGGATCCAAGAAGAGGATGTTCTCCTGAGGAAATGCATTGAGAAATAT
HU        AGAAAGGGTGCATGGATCCAAGAAGAGGATGTTCTCCTGAGGAAATGCATTGAGAAATAT
Vshi3     AGAAAGGGTGCATGGACCCAAGAAGAGGATGCTCTCCTGAGGAAATGCATTGAGAAATAT
Vsa2      AGAAAGGGTGCATGGACCCAAGAAGAGGATGTTCTCCTGAGGAAATGCATTGAGAAATAT
Vshi2     AGAAAGGGTGCATGGACCCAAGAAGAGGATGTTCTCCTGAGGAAATGCATTGAGAAATAT
Vfi2      AGAAAGGGTGCATGGATCCAAGAAGAGGATGTTCTCCTGAGGAAATGCATTGACAAATAT
CS        AGAAAGGGTGCATGGATCCAAGAAGAGGATGTTCTCCTGAGGAAATGCATTGAGAAATAT
Syr       AGAAAGGGTGCATGGATCCAAGAAGAGGATGTTCTCCTGAGGAAATGCATTGAGAAATAT
PN        AGAAAGGGTGCATGGATCCAAGAAGAGGATGTTCTCCTGAGGAAATGCATTGAGAAATAT
Me        AGAAAGGGTGCATGGATCCAAGAAGAGGATGTTCTCCTGAGGAAATGCATTGAGAAATAT
Vco2      AGAAAGGGTGCATGGATCCAAGAAGAGGATGTTCTCCTGAGGAAATGCATTGAGAAATAT
Vamu2     AGAAAGGGTGCATGGATCCAAGAAGAGGATGTTCTCCTGAGGAAATGCATTGAGAAATAT
Vlcon     AGAAAGGGTGCATGGATCCAAGAAGAGGATGTTCTCCTGAGGAAATGCGTTGAGAAATAT
Vru       AGAAAGGGTGCATGGATCCAAGAAGAGGATGTTCTCCTGAGGAAATGCATTGAGAAATAT
Vri       AGAAAGGGTGCATGGATCCAAGAAGAGGATGTTCTCCTGAGGAAATGCATTGAGAAATAT
Vldela    AGAAAGGGTGCATGGATCCAAGAAGAGGATGTTCTCCTGAGGAAATGCATTGAGAAATAT
Vco1      AGAAAGGGTGCATGGATCCAAGAAGAGGATGTTCTCCTGAGGAAATGCATTGAGAAATAT
Vamu1     AGAAAGGGTGCATGGATCCAAGAAGAGGATGTTCTCCTGAGGAAATGCATTGAGAAATAT
Vfi1      AGAAAGGGTGCATGGATCCAAGAAGAGGATGTTCTCCTGAGGAAATGCATTGACAAATAT
Vshi1     AGAAAGGGTGCATGGATCCAAGAAGAGGATGTTCTCCTGAGGAAATGCATTGACAAATAT
Vsa1      AGAAAGGGTGCATGGATCCAAGAAGAGGATGTTCTCCTGAGGAAATGCATTGACAAATAT
          ********* ****** ************** **************** **** ******


Vfl       GGAGAAGGAAAGTGGCATCTGGTTCCCCTCCGAGCAGGTAACATAAAAGAGAAAGGGATC
KS        GGAGAAGGAAAGTGGCATCTGGTTCCCCTCCGAGCAGGTAACATGAAAGAGAAAGGGATC
Ryu       GGAGAAGGAAAGTGGCATCTGGTTCCCCTCCGAGCAGGTAACATGAAAGAGAAAGGGATC
HU        GGAGAAGGAAAGTGGCATCTGGTTCCCCTCCGAGCAGGTAACATGAAAGAGAAAGGGATC
Vshi3     GGAGAAGGAAAGTGGCATCTGGTTCCCCTCCGAGCAGGTAACATGAAAGAGAAAGGGATC
Vsa2      GGAGAAGGAAAGTGGCATCTGGTTCCCCTCCGAGCAGGTAACATGAAAGAGAAAGGGATC
Vshi2     GGAGAAGGAAAGTGGCATCTGGTTCCCCTCCGAGCAGGTAACATGAAAGAGAAAGGGATC
Vfi2      GGAGAAGGAAAGTGGCATCTGGTTCCCCTCCGAGCAGGTAACATGAAAGAGAAAGGGATC
CS        GGAGAAGGAAAGTGGCATCTGGTTCCCCTCCGAGCAGGTAACATGAAAGAGAAAGGGATC
Syr       GGAGAAGGAAAGTGGCATCTGGTTCCCCTCCGAGCAGGTAACATGAAAGAGAAAGGGATC
PN        GGAGAAGGAAAGTGGCATCTGGTTCCCCTCCGAGCAGGTAACATGAAAGAGAAAGGGATC
Me        GGAGAAGGAAAGTGGCATCTGGTTCCCCTCCGAGCAGGTAACATGAAAGAGAAAGGGATC
Vco2      GGAGAAGGAAAGTGGCATCTGGTTCCCCTCCGAGCAGGTAACATGAAAGAGAAAGGGATC
Vamu2     GGAGAAGGAAAGTGGCATCTGGTTCCCCTCCGAGCAGGTAACATGAAAGAGAAAGGGATC
Vlcon     GGAGAAGGAAAGTGGCATCTGGTTCCCCTCCGAGCAGGTAACATGAAAGAGAAAGGGATC
Vru       GGAGAAGGAAAGTGGCATCTGGTTCCCCTCCGAGCAGGTAACATGAAAGAGAAAGGGATC
Vri       GGAGAAGGAAAGTGGCATCTGGTTCCCCTCCGAGCAGGTAACATGAAAGAGAAAGGGATC
Vldela    GGAGAAGGAAAGTGGCATCTGGTTCCCCTCCGAGCAGGTAACATGAAAGAGAAAGGGATC
Vco1      GGAGAAGGAAAGTGGCATCTGGTTCCCCTCCGAGCAGGTAACATGAAAGAGAAAGGGGTC
Vamu1     GGAGAAGGAAAGTGGCATCTGGTTCCCCTCCGAGCAGGTAACATGAAAGAGAAAGGGGTC
Vfi1      GGAGAAGGAAAGTGGCATCTGGTTCCCCTCCGAGCAGGTAACATGAAAGAGAAATGGATC
Vshi1     GGAGAAGGAAAGTGGCATCTGGTTCCCCTCCGAGCAGGTAACATGAAAGAGAAATGGATC
Vsa1      GGAGAAGGAAAGTGGCATCTGGTTCCCCTCCGAGCAGGTAACATGAAAGAGAAATGGATC
          ******************************************** ********* ** **
```

```
Vfl         AGTATTAATTTGTGTTTTTTTACTTCTGTTTT------------GCTTAAAGAGTTTCGT
KS          AGTATTAATTTGTGTTTTATTACTTCTGTTTT------------GCTTAAAGAGTTTCGT
Ryu         AGTATTAATTTGTGTTTTATTACTTCTGTTTT------------GCTTAAAGAGTTTCGT
HU          AGTATTAATTTGTGTTTTATTACTTCTGTTTT------------GCTTAAAGAGTTTCGT
Vshi3       AGTATTAATTTGTGTTTTTTTACTTCTGTTTT------------GCTTAAAGAGTTTCGT
Vsa2        AGTATTAATTTGTGTTTTTTTACTTCTGTTTT------------GCTTAAAGAGTTTCGT
Vshi2       AGTATTAATTTGTGTTTTTTTACTTCTGTTTT------------GCTTAGAGAGTTTCGT
Vfi2        AGTATTAATTTGTGTTTTTTTACTTCTGTTTT------------GCTTAAAGAGTTTCGT
CS          AGTATTTATTTGTGTTTTTTTACTTCTGTTTT------------GCTTAAAGAGTTTCAT
Syr         AGTATTTATTTGTGTTTTTTTACTTCTGTTTT------------GCTTAAAGAGTTTCAT
PN          AGTATTTATTTGTGTTTTTTTACTTCTGTTTT------------GCTTAAAGAGTTTCAT
Me          AGTATTTATTTGTGTTTTTTTACTTCTGTTTT------------GCTTAAAGAGTTTCAT
Vco2        AGTATTAATTTGTGTTTTTTTACTTCTGTTTT------------GCTTAAAGAGTTTCAT
Vamu2       AGTATTAATTTGTGTTTTTTTACTTCTGTTTT------------GCTTAAAGAGTTTCAT
Vlcon       AGTATTAATTTGTGTTTTTTTACTTCTGTTTT------------GCTTAAAGAGTTTCGT
Vru         AGTATTAATTTGTGTTTTTTTACTTCTGTTTT------------GCTTAGAGAGTTTCGT
Vri         AGTATTAATTTGTGTTTTTTTACTTCTGTTTT------------GCTTAGAGAGTTTCGT
Vldela      AGTATTAATTTGTGTTTTTTTACTTCTGTTTT------------GCTTAGAGAGTTTCGT
Vco1        AGTATTAATTTGTGTTTTTTTACTTCTGTTTT------------GCTTAAAGAGTTTCGT
Vamu1       AGTATTAATTTGTGTTTTTTTACTTCTGTTTT------------GCTTAAAGAGTTTCGT
Vfi1        ACTATTAATTTGTGTGTGTGTGTTTTTTTTTT-ACTTCTGCTTTGCTTAAAGAGTTTCGT
Vshi1       ACTATTAATTTGTGTGTGTGTGTTTT------------------GCTTAAAGAGCTTCGT
Vsa1        AGTACTAATTTGTGTGTGTGTTTTTTTTTTTTTACTTCTGTTTTGCTTAAAGAGTTTCGT
            * ** * ******** *   *  **                   ***** **** *** *


Vfl         TTTCTTGAGTTTGCAGGGTTGAATAGATGCCGAAAAAGCTGCAGGTTGAGATGGCTCAAT
KS          TTTCTTGAGTTTGCAGGGTTGAATAGATGCCGAAAAAGCTGCAGATTGAGATGGCTCAAT
Ryu         TTTCTTGAGTTTGCAGGGTTGAATAGATGCCGAAAAAGCTGCAGATTGAGATGGCTCAAT
HU          TTTCTTGAGTTTGCAGGGTTGAATAGATGCCGAAAAAGCTGCAGATTGAGATGGCTCAAT
Vshi3       TTTCTTGAGTTTGCAGGGTTGAATAGATGCCGAAAAAGCTGCAGGTTGAGATGGCTCAAT
Vsa2        TTTCTTGAGTTTGCAGGGTTGAATAGATGCCGAAAAAGCTGCAGGTTGAGATGGCTCAAT
Vshi2       TTTCTTGAGTTTTCAGGGTTGAATAGATGCCGAAAAAGCTGCAGATTGAGATGGCTCAAT
Vfi2        TTTCTTGAGTTTGCAGGGTTGAATAGATGCCGAAAAAGCCGCAGGTTGAGATGGCTCAAT
CS          TTTCTTGAGTTTGCAGGGTTGAATAGATGCCGAAAAAGCTGCAGGTTGAGATGGCTCAAT
Syr         TTTCTTGAGTTTGCAGGGTTGAATAGATGCCGAAAAAGCTGCAGGTTGAGATGGCTCAAT
PN          TTTCTTGAGTTTGCAGGGTTGAATAGATGCCGAAAAAGCTGCAGGTTGAGATGGCTCAAT
Me          TTTCTTGAGTTTGCAGGGTTGAATAGATGCCGAAAAAGCTGCAGGTTGAGATGGCTCAAT
Vco2        TTTCTTGAGTTTGCAGGGTTGAATAGATGCCGAAAAAGCTGCAGGTTGAGATGGCTCAAT
Vamu2       TTTCTTGAGTTTGCAGGGTTGAATAGATGCCGAAAAAGCTGCAGGTTGAGATGGCTCAAT
Vlcon       TTTCTTGAGTTTGCAGGGTTGAATAGATGCCGAAAAAGCTGCAGGTTGAGATGGCTCAAT
Vru         TTTCTTGAGTTTGCAGGGTTGAATAGATGCCGAAAAAGCTGCAGGTTGAGATGGCTCAAT
Vri         TTTCTTGAGTTTGCAGGGTTGAATAGATGCCGAAAAAGCTGCAGGTTGAGATGGCTCAAT
Vldela      TTTCTTGAGTTTGCAGGGTTGAATAGATGCCGAAAAAGCTGCAGGTTGAGATGGCTCAAT
Vco1        TTTCTTGAGTTTGCAGGGTTGAATAGATGCCGAAAAAGCTGCAGGTTGAGATGGCTCAAT
Vamu1       TTTCTTGAGTTTGCAGGGTTGAATAGATGCCGAAAAAGCTGCAGGTTGAGATGGCTCAAT
Vfi1        TTTCTTGAGTTTGCAGGGTTGAATAGATGCCGAAAAAGCTGCAGGTTGAGATGGCTCAAT
Vshi1       TTCCTTGAGTTTGCAGGGTTGAATAGATGCCGAAAAAGCTGCAGGTTGAGATGGCTCAAT
Vsa1        TTTCTTGAGTTTGCAGGGTTGAATAGATGCCGAAAAAGCTGCAGGTTGAGATGGCTCAAT
            ** ********** ************************* **** ***************
```

```
Vfl        TATTTGAAGCCGGATATCAAGAGAGGAGAGTTTGCATTAGACGAGGTTGATCTCATGATT
KS         TATTTGAAGCCGGATATCAAGAGAGGAGAGTTTGCATTAGACGAGGTTGACCTCATGATT
Ryu        TATTTGAAGCCGGATATCAAGAGAGGAGAGTTTGCATTAGACGAGGTTGACCTCATGATT
HU         TATTTGAAGCCGGATATCAAGAGAGGAGAGTTTGCATTAGACGAGGTTGACCTCATGATT
Vshi3      TATTTGAAGCCGGATATCAAGAGAGGAGAGTTTGCATTAGACGAGGTTGATCTCATGATT
Vsa2       TATTTGAAGCCGGATATCAAGAGAGGAGAGTTTGCATTAGACGAGGTTGATCTCATGATT
Vshi2      TATTTGAAGCCAGATATCAAGAGAGGGGAGTTTGCATTAGACGAGGTTGATCTCATGATT
Vfi2       TATTTGAAGCCGGATATCAAGAGAGGAGAGTTTGCATTAGACGAGGTTGATCTCATGATT
CS         TATTTGAAGCCGGATATCAAGAGAGGAGAGTTTGCATTAGACGAGGTTGATCTCATGATT
Syr        TATTTGAAGCCGGATATCAAGAGAGGAGAGTTTGCATTAGACGAGGTTGATCTCATGATT
PN         TATTTGAAGCCGGATATCAAGAGAGGAGAGTTTGCATTAGACGAGGTTGATCTCATGATT
Me         TATTTGAAGCCGGATATCAAGAGAGGAGAGTTTGCATTAGACGAGGTTGATCTCATGATT
Vco2       TATTTGAAGCCGGATATCAAGAGAGGAGAGTTTGCATTAGACGAGGTTGATCTCATGATT
Vamu2      TATTTGAAGCCGGGTATCAAGAGAGGAGAGTTTGCATTAGACGAGGTTGATCTCATGATT
Vlcon      TATTTGAAGCCGGATATCAAGAGAGGAGAGTTTGCATTAGACGAGGTTGATCTCATGATT
Vru        TATTTGAAGCCGGATATCAAGAGAGGAGAGTTTGCATTAGACGAGGTTGATCTCATGATT
Vri        TATTTGAAGCCGGGTATCAAGAGAGGAGAGTTTGCATTAGACGAGGTTGATCTCATGATT
Vldela     TATTTGAAGCCGGATATCAAGAGAGGAGAGTTTGCATTAGACGAGGTTGATCTCATGATT
Vco1       TATTTGAAGCCGGATATCAAGAGAGGAGAGTTTACATTAGACGAGGTTGATCTCATGATT
Vamu1      TATTTGAAGCCGGATATCAAGAGAGGAGAGTTTACATTAGACGAGGTTGATCTCATGATT
Vfi1       TATTTGAAGCCGGATATCAAGAGAGGAGAGTTTGCATTAGACGAGGTTGATCTCATGATT
Vshi1      TATTTGAAGCCGGATATCAAGAGAGGAGAGTTTGCATTAGACGAGGTTGATCTCATGATT
Vsa1       TATTTGAAGCCGGATATCAAGAGAGGAGAGTTTGCATTAGACGAGGTTGATCTCATGATT
           *********** * ************ ****** **************** *********

Vfl        AGGCTTCACAATTTGTTGGGGAACAGGCAAGTCTATGATAACTCAAGTACTAGCTTAATA
KS         AGGCTTCACAATTTGTTGGGGAACAGGCAAGTCTATAATAACTCAAGTACTAGCTTGATA
Ryu        AGGCTTCACAATTTGTTGGGGAACAGGCAAGTCTATAATAACTCAAGTACTAGCTTGATA
HU         AGGCTTCACAATTTGTTGGGGAACAGGCAAGTCTATAATAACTCAAGTACTAGCTTGATA
Vshi3      AGGCTTCACAATTTGTTGGGGAACAGGCAAGTCTATAATAACTCAAGTACTAGCTTGATA
Vsa2       AGGCTTCACAATTTGTTGGGGAACAGGCAAGTCTATAATAACTCAAGTACTAGCTTGATA
Vshi2      AGGCTTCACAATTTGTTGGGGAACAGGCAAGTCTATAATAACTCAAGTACTAGCTTGATA
Vfi2       AGGCTTCACAATTTGTTGGGGAACAGGCAAGTCTATAATAACTCAAGTACTAGCTTGATA
CS         AGGCTTCACAATTTGTTGGGGAACAGGCAAGTCTATAATAACTCAAGTACTAGCTTGATA
Syr        AGGCTTCACAATTTGTTGGGGAACAGGCAAGTCTATAATAACTCAAGTACTAGCTTGATA
PN         AGGCTTCACAATTTGTTGGGGAACAGGCAAGTCTATAATAACTCAAGTACTAGCTTGATA
Me         AGGCTTCACAATTTGTTGGGGAACAGGCAAGTCTATAATAACTCAAGTACTAGCTTGATA
Vco2       AGGCTTCACAATTTGTTGGGGAACAGGCAAGTCTATAATAACTCAAGTACTAGCTTGATA
Vamu2      AGGCTTCACAATTTGTTGGGGAACAGGCAAGTCTATAATAACTCAAGTACTAGCTTGATA
Vlcon      AGGCTTCACAATTTGTTGGGGAACAGGCAAGTCTATAATAACTCAAGTACTAGCTTGATA
Vru        AGGCTTCACAATTTGTTGGGGAACAGGCAAGTCTATAATAACTCAAGTACTAGCTTGATA
Vri        AGGCTTCACAATTTGTTGGGGAACAGGCAAGTCTATAATAACTCAAGTACTAGCTTGATA
Vldela     AGGCTTCACAATTTGTTGGGGAACAGGCAAGTCTATAATAACTCAAGTACTAGCTTGATA
Vco1       AGGCTTCACAATTTGTTGGGGAACAGGCAAGTCTATAATAACTCAAGTACTAGCTTGATA
Vamu1      AGGCTTCACAATTTGTTGGGGAACAGGCAAGTCTATAATAACTCAAGTACTAGCTTGATA
Vfi1       AGGCTTCACAATTTGTTGGGGAACAGGCAAGTCTATAATAACTCAAGTACTAGCTTGATA
Vshi1      AGGCTTCACAATTTGTTGGGGAACAGGCAAGTCTATAATAACTCAGGTACTAGCTTGATA
Vsa1       AGGCTTCACAATTTGTTGGGGAACAGGCAAGTCTATAATAACTCAAGTATTAGCTTGATA
           ************************************ ******** *** ****** ***
```

```
Vfl       ATGATATTATATTAGTTCTGAAGCTGTTCAGAACTTACAAAAGA----------------
KS        ATGATATTATATTAGTTCTGAAGCTGTTCAGAACTTACAAATTAGAAAAGCCCCCATGAA
Ryu       ATGATATTATATTAGTTCTGAAGCTGTTCAGAACTTACAAATTAGAAAAGCCCCCATGAA
HU        ATGATATTATATTAGTTCTGAAGCTGTTCAGAACTTACAAATTAGAAAAGCCCCCATGAA
Vshi3     ATGATATTATATTAGTTCTGAAGCTGTTCAG-----------------------------
Vsa2      ATGATATTATATTAGTTCTGAAGCTGTTCAG-----------------------------
Vshi2     ATGATATTATATTAGTTCTGAAGCTGTTCAGAACTTACAAAAGA----------------
Vfi2      ATGATATTATATTAGTTCTGAAGCTGTTCAGAACTTACAAAAGA----------------
CS        ATGATATTATATTAGTTCTGAAGCTGTTCAGAACTTACAAAAGA----------------
Syr       ATGATATTATATTAGTTCTGAAGCTGTTCAGAACTTACAAAAGA----------------
PN        ATGATATTATATTAGTTCTGAAGCTGTTCAGAACTTACAAAAGA----------------
Me        ATGATATTATATTAGTTCTGAAGCTGTTCAGAACTTACAAAAGA----------------
Vco2      ATGATATTATATTAGTTCTGAAGCTGTTCAGAACTTACAAAAGA----------------
Vamu2     ATGATATTATATTAGTTCTGAAGCTGTTCAGAACTTACAAAAGA----------------
Vlcon     ATGATATTATATTAGTTCTGAAGCTGTTCAGAACTTACAAAAGA----------------
Vru       ATGATATTATATTAGTTCTGAAGCTGTTCAGAACTTTCAAAAGA----------------
Vri       ATGATATTATATTAGTTCTGAAGCTGTTCAGAACTTTCAAAAGA----------------
Vldela    ATGATATTATATTAGTTCTGAAGCTGTTCAGAACTTACAAAAGA----------------
Vco1      ATGATATTATATTAGTTCTGAAGCTGTTCAGAACTTACAAAAGA----------------
Vamu1     ATGATATTATATTAGTTCTGAAGCTGTTCAGAACTTACAAAAGA----------------
Vfi1      ATGATATTATATTAGTTCTGAAGCTGTTCAG-----------------------------
Vshi1     ATGATATTATATTAGTTCTGAAGCTGTTCAG-----------------------------
Vsa1      ATGATATTATATTAGTTCTGAAGCTGTTCAG-----------------------------
          *******************************

Vfl       -----------------GCTGTTCAGTTGATACTTTGTCTGATGTTGTGCGTGTATAGAT
KS        TTAGAACTTACAAAAGAGCTGTTCAGTTGATACTTTGTCTGATGTTGTGCGTGTATAGAT
Ryu       TTAGAACTTACAAAAGAGCTGTTCAGTTGATACTTTGTCTGATGTTGTGCGTGTATAGAT
HU        TTAGAACTTACAAAAGAGCTGTTCAGTTGATACTTTGTCTGATGTTGTGCGTGTATAGAT
Vshi3     --------------------------TTGATACTTTGTCTGATGTTGTGCGTGTATAGAT
Vsa2      --------------------------TTGATACTTTGTCTGATGTTGTGCGTGTATAGAT
Vshi2     -----------------GCTGTTCAGTTGATACTTTGTCTGATGTTGTGCGTGTATAGAT
Vfi2      -----------------GCTGTTCAGTTGATACTTTGTCTGATGTTGTGCGTGTATAGAT
CS        -----------------GCTGTTCAGTTGATACTTTGTCTGATGTTGTGCGTGTATAGAT
Syr       -----------------GCTGTTCAGTTGATACTTTGTCTGATGTTGTGCGTGTATAGAT
PN        -----------------GCTGTTCAGTTGATACTTTGTCTGATGTTGTGCGTGTATAGAT
Me        -----------------GCTGTTCAGTTGATACTTTGTCTGATGTTGTGCGTGTATAGAT
Vco2      -----------------GCTGTTCAGTTGATACTTTGTCTGATGTTGTGCGTGTATAGAT
Vamu2     -----------------GCTGTTCAGTTGATACTTTGTCTGATGTTGTGCGTGTATAGAT
Vlcon     -----------------GCTGTTCAGTTGATACTTTGTCTGATGT---GCGTGTATAGAT
Vru       -----------------GCTGATCAGTTGATGCTTTGTCTGATGTTGTGCGTGTATAGAT
Vri       -----------------GCTGATCAGTTGATACTTTGTCTGATGTTGTGCGTGTATAGAT
Vldela    -----------------GCTGTTCAGTTGATACTTTGTCTGATGTTGTGCGTGTATAGAT
Vco1      -----------------GCTGTTCAGTTGATACTTTGTCTGATGTTGTGCGTGTATAGAT
Vamu1     -----------------GCTGTTCAGTTGATACTTTGTCTGATGTTGTGCGTGTATAGAT
Vfi1      --------------------------TTGATACTTTGTCTGATGTTGTGCGTGTATAGAT
Vshi1     --------------------------TTGATACTTTGTCTGATGTTGTGCCTGTATAGAT
Vsa1      --------------------------TTGATACTTTGTCTGATGTTGTGCGTGTATAGAT
                                    ***** *************   ** *********
```

```
Vfl        GGTCCTTGATTGCGGGTAGGCTTCCTGGGAGGACTGCTAATGATGTCAAGAACTATTGGC
KS         GGTCCTTGATTGCGGGTAGGCTTCCAGGGAGGACTGCTAATGATGTCAAGAACTATTGGC
Ryu        GGTCCTTGATTGCGGGTAGGCTTCCAGGGAGGACTGCTAATGATGTCAAGAACTATTGGC
HU         GGTCCTTGATTGCGGGTAGGCTTCCAGGGAGGACTGCTAATGATGTCAAGAACTATTGGC
Vshi3      GGTCCTTGATTGCGGGTAGGCTTCCAGGGCGGACTGCTAATGATGTCAAGAACTATTGGC
Vsa2       GGTCCTTGATTGCGGGTAGGCTTCCAGGGAGGACTGCTAATGATGTCAAGAACTATTGGC
Vshi2      GGTCCTTGATTGCGGGTAGGCTTCCAGGGAGGACTGCTAATGATGTCAAGAACTATTGGC
Vfi2       GGTCCTTGATTGCGGGTAGGCTTCCAGGGAGGACTGCTAATGATGTCAAGAACTATTGGC
CS         GGTCCTTGATTGCGGGTAGGCTTCCAGGGAGGACTGCCAATGATGTCAAGAACTATTGGC
Syr        GGTCCTTGATTGCGGGTAGGCTTCCAGGGAGGACTGCTAATGATGTCAAGAACTATTGGC
PN         GGTCCTTGATTGCGGGTAGGCTTCCAGGGAGGACTGCTAATGATGTCAAGAACTATTGGC
Me         GGTCCTTGATTGCGGGTAGGCTTCCAGGGAGGACTGCTAATGATGTCAAGAACTATTGGC
Vco2       GGTCCTTGATTGCGGGTAGGCTTCCAGGGAGGACTGCTAATGATGTCAAGAACTATTGGC
Vamu2      GGTCCTTGATTGCGGGTAGGCTTCCAGGGAGGACTGCTAATGATGTCAAGAACTATTGGC
Vlcon      GGTCCTTGATTGCGGGTAGGCTTCCAGGGAGGACTGCTAATGATGTCAAGAACTATTGGC
Vru        GGTCCTTGATTGCTGGTAGGCTTCCAGGGAGGACTGCTAATGATGTCAAGAACTATTGGC
Vri        GGTCCTTGATTGCTGGTAGGCTTCCAGGGAGGACTGCTAATGATGTCAAGAACTATTGGC
Vldela     GGTCCTTGATTGCGGGTAGGCTTCCAGGGAGGACTGCTAATGATGTCAAGAACTATTGGC
Vco1       GGTCCTTGATTGCGGGTAGGCTTCCAGGGAGGACTGCTAATGATGTCAAGAACTATTGGC
Vamu1      GGTCCTTGATTGCGGGTAGGCTTCCAGGGAGGACTGCTAATGATGTCAAGAACTATTGGC
Vfi1       GGTCCTTGATTGCGGGTAGGCTTCCAGGGAGGACTGCTAATGATGTCAAGAACTATTGGC
Vshi1      GGTCCTTGATTGCGGGTAGGCTTCCAGGGCGGACTGCTAATGATGTCAAGAACTATTGGT
Vsa1       GGTCCTTGATTGCGGGTAGGCTTCCAGGGAGGACTGCTAATGATGTCAAGAACTATTGGC
           ************* *********** *** ******* ********************* 

Vfl        ATAGTCACCACTTCAAAAAGGAGGTTC
KS         ATAGTCACCACTTCAAAAAGGAGGTTC
Ryu        ATAGTCACCACTTCAAAAAGGAGGTTC
HU         ATAGTCACCACTTCAAAAAGGAGGTTC
Vshi3      ATAGTCACCACTTCAAAAAGGAGGTTC
Vsa2       ATAGTCACCACTTCAAAAAGGAGGTTC
Vshi2      ATAGTCACCACTTCAAAAAGGAGGTTC
Vfi2       ATAGTCACCACTTCAAAAAGGAGGTTC
CS         ATAGTCACCACTTCAAAAAGGAGGTTC
Syr        ATAGTCACCACTTCAAAAAGGAGGTTC
PN         ATAGTCACCACTTCAAAAAGGAGGTTC
Me         ATAGTCACCACTTCAAAAAGGAGGTTC
Vco2       ATAGTCACCACTTCAAAAAGGAGGTTC
Vamu2      ATAGTCACCACTTCAAAAAGGAGGTTC
Vlcon      ATAGTCACCACTTCAAAAAGGAGGTTC
Vru        ATAGTCACCACTTCAAAAAGGAGGTTC
Vri        ATAGTCACCACTTCAAAAAGGAGGTTC
Vldela     ATAGTCACCACTTCAAAAAGGAGGTTC
Vco1       ATAGTCACCACTTCAAAAAGGAGGTTC
Vamu1      ATAGTCACCACTTCAAAAAGGAGGTTC
Vfi1       ATAGTCACCACTTCAAAAAGGAGGTTC
Vshi1      ATGGTCACCACTTCAAAAAGGAGGTTC
Vsa1       ATAGTCACCACTTCAAAAAGGAGGTTC
           ** ************************
```

Figure S3. Alignment of nucleotide sequences of *MybA1* genes of the red allele among grapes. The 33 bp short fragments in the second intron of the *MybA1* gene of oriental *V. vinifera* cultivars are shaded black. Identical nucleotides are indicated by asterisks. Vfl, *V. flexuosa*. KS, Koshu. Ryu, Ryugan. HU, Huotianhong. Vshi3, *V. shiragai* red allele 3. Vsa2, *V. saccharifera* red allele 2. Vshi2, *V. shiragai* red allele 2. Vfi2, *V. ficifolia* red allele 2. CS, Cabernet Sauvignon. Syr, Syrah. PN, Pinot Noir. Me, Merlot. Vco2, *V. coignetiae* red allele 2. Vamu2, *V. amurensis* red alelle 2. Vlcon, *V. labrusca* cv. Concord. Vru, *V. rupestris*. Vri, *V. riparia*. Vldela, *V. labruscana* cv. Delaware. Vco1, *V. coignetiae* red allele 1. Vamu1, *V. amurensis* red alelle 1. Vfi1, *V. ficifolia* red allele 1. Vshi1, *V. shiragai* red allele 2. Vsa1, *V. saccharifera* red allele 1.
