# Supplementary material for: Pink-Colored Grape Berry Is the Result of Short Insertion in Intron of Color Regulatory Gene
Source: PLoS One. 2011 Jun 17;6(6):e21308. doi: 10.1371/journal.pone.0021308 (PMC3117884; doi:10.1371/journal.pone.0021308)
Supplement: Figure S4 — Phylogenetic tree of MybA1 genes of the red allele among grapes. Bootstrap values are indicated on the branches. CS, Cabernet Sauvignon. ME, Merlot. SYR, Syrah. PN, Pinot Noir. KS, Koshu. RYU, Ryugan. HU, Huotianhong. (PDF) [file pone.0021308.s004.pdf]

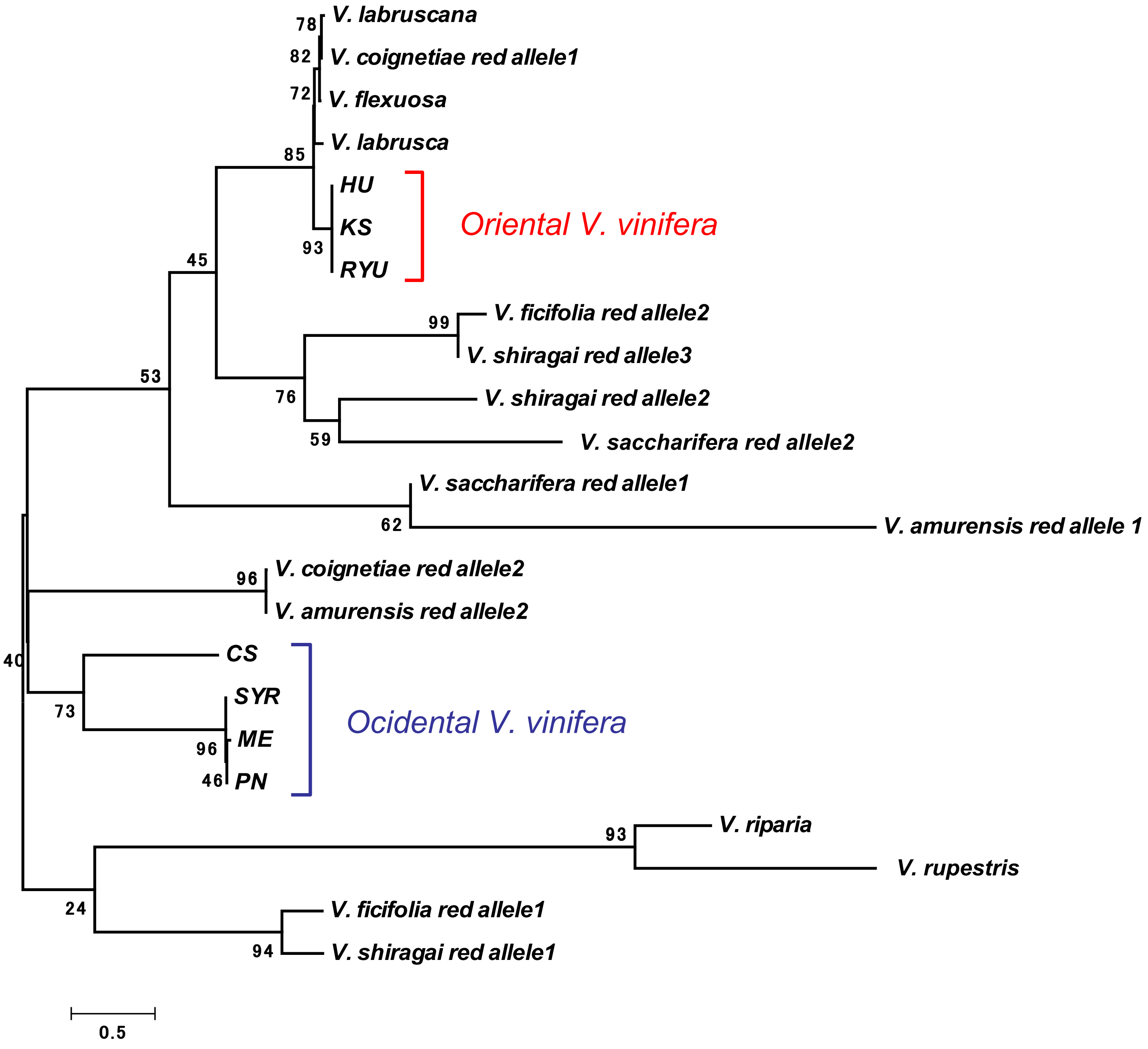

Figure S4. Phylogenetic tree of *MybA1* genes of the red allele among grapes. Bootstrap values are indicated on the branches. CS, Cabernet Sauvignon. ME, Merlot. SYR, Syrah. PN, Pinot Noir. KS, Koshu. RYU, Ryugan. HU, Huotianhong.
