# Supplementary material for: Pink-Colored Grape Berry Is the Result of Short Insertion in Intron of Color Regulatory Gene
Source: PLoS One. 2011 Jun 17;6(6):e21308. doi: 10.1371/journal.pone.0021308 (PMC3117884; doi:10.1371/journal.pone.0021308)
Supplement: Table S1 — Size of MybA1 PCR products amplified from red alleles in Vitis species. (PDF) [file pone.0021308.s005.pdf]

Table S1 Size of *MybA1* PCR products amplified from red alleles in *Vitis* species

species	cultivar	size (bp)	accession no.	skin color
V. vinifera				
occidentalis	Cabernet Sauvignon	845	GU145119	black
	Merlot	846	GU145120	black
	Syrah	846	GU145121	black
	Pinot Noir	846	GU145122	black
orientalis	Koshu	1035	GQ890360	pink
	Ryugan	1035	GU145112	pink
	Huotianhong	1035	GU145113	pink
North Ammerica				
V. labrusca	Concord	999	GU145123	black
V. labruscana	Delaware	1002	GU145124	rose
V. rupestris	St. George	968	GU145125	black
V. riparia	Gloire de Montpellier	967	GU145126	black
East Asia				
V. ficifolia		972	GU145127	black
		891	GU145128	
V. coignetiae		1002	GU145129	black
		847	GU145130	
V. flexuosa		1002	GU145131	black
V. shiragai		955	GU145132	black
		890	GU145133	
		869	GU145134	
V. saccharifera		991	GU145115	black
		867	GU145116	
V. amurensis		1001	GU145117	black
		847	GU145118	
